# Vibriosis Outbreaks in Aquaculture: Addressing Environmental and Public Health Concerns and Preventive Therapies Using Gilthead Seabream Farming as a Model System

**DOI:** 10.3389/fmicb.2022.904815

**Published:** 2022-07-11

**Authors:** Gracinda M. M. Sanches-Fernandes, Isabel Sá-Correia, Rodrigo Costa

**Affiliations:** ^1^Institute for Bioengineering and Biosciences, Biological Sciences Research Group, Instituto Superior Técnico, Universidade de Lisboa, Lisbon, Portugal; ^2^Department of Bioengineering, Instituto Superior Técnico, Universidade de Lisboa, Lisbon, Portugal; ^3^Associate Laboratory i4HB—Institute for Health and Bioeconomy at Instituto Superior Técnico, Universidade de Lisboa, Lisbon, Portugal; ^4^Centre of Marine Sciences, University of Algarve, Faro, Portugal

**Keywords:** biological control, fish larviculture, fish microbiome, host-microbe interactions, probiotics, *Vibrio*

## Abstract

Bacterial and viral diseases in aquaculture result in severe production and economic losses. Among pathogenic bacteria, species belonging to the *Vibrio* genus are one of the most common and widespread disease-causing agents. *Vibrio* infections play a leading role in constraining the sustainable growth of the aquaculture sector worldwide and, consequently, are the target of manifold disease prevention strategies. During the early, larval stages of development, *Vibrio* species are a common cause of high mortality rates in reared fish and shellfish, circumstances under which the host organisms might be highly susceptible to disease preventive or treatment strategies such as vaccines and antibiotics use, respectively. Regardless of host developmental stage, *Vibrio* infections may occur suddenly and can lead to the loss of the entire population reared in a given aquaculture system. Furthermore, the frequency of *Vibrio*–associated diseases in humans is increasing globally and has been linked to anthropic activities, in particular human-driven climate change and intensive livestock production. In this context, here we cover the current knowledge of *Vibrio* infections in fish aquaculture, with a focus on the model species gilthead seabream (*Sparus aurata*), a highly valuable reared fish in the Mediterranean climatic zone. Molecular methods currently used for fast detection and identification of *Vibrio* pathogens and their antibiotic resistance profiles are addressed. Targeted therapeutic approaches are critically examined. They include vaccination, phage therapy and probiotics supplementation, which bear promise in supressing vibriosis in land-based fish rearing and in mitigating possible threats to human health and the environment. This literature review suggests that antibiotic resistance is increasing among *Vibrio* species, with the use of probiotics constituting a promising, sustainable approach to prevent *Vibrio* infections in aquaculture.

## Introduction

### Aquaculture Production and Commercial Value

**Figure 1:**
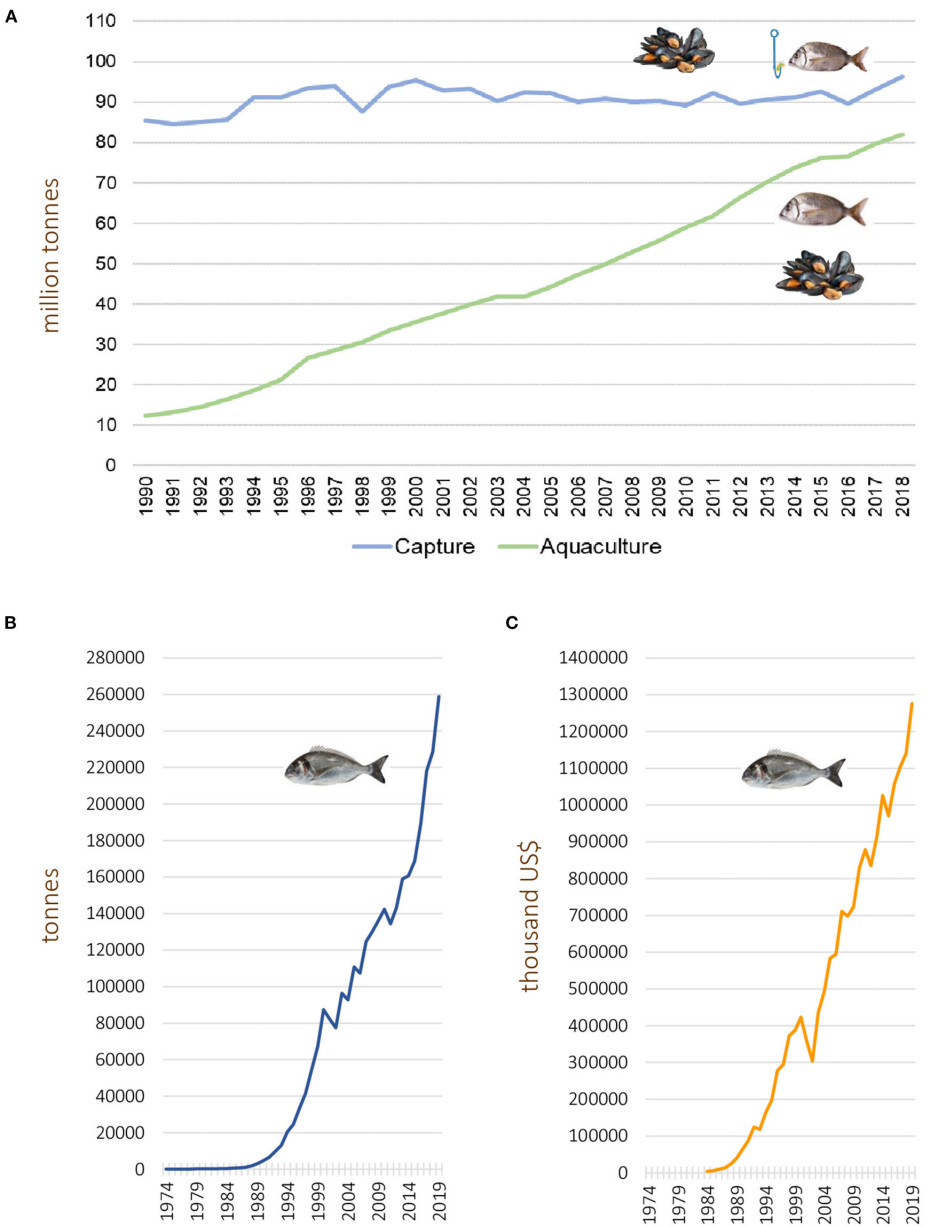
**(A)** Evolution of wild capture and aquaculture-based seafood production (million tonnes, live weight). The total seafood production is growing due to the increase in fish / shellfish biomass derived from the aquaculture sector, contrasting with near constant capture values. The data include fish, crustaceans, molluscs, and other cultured aquatic animals, and were retrieved from reports by the Food and Agriculture Organization of the United Nations (FAO) spanning the period (FAO, 1996, 2002, 2004, 2010, 2012, 2016, 2018, 2020). **(B,C)** show the worldwide gilthead seabream aquaculture production (in tonnes) **(B)** and their commercial value (thousand US$) **(C)**. Data collected from FAO, query online, http://www.fao.org/fishery/statistics/global-aquaculture-production/query/en.

Global seafood production including fish, crustaceans, molluscs, and other aquatic animals but excluding aquatic mammals, reptiles, seaweeds, and other aquatic plants, was estimated to reach 179 million tonnes in 2018, with an approximate first sale value of 401 billion US$, being the aquaculture sector responsible for 250 billion US$. This corresponds to a worldwide production of 82.1 million tonnes derived from aquaculture practices compared with 96 million tonnes from wild captures (FAO, 2020) (Figure 1A). Totals of 22.2 million tonnes were used, in that year, for fish meal and fish oil production, while 156.4 million tonnes were used for human consumption, matching the demand for seafood production by the growing human population, which reached a record high of 20,5 kg per capita (FAO, 2020). Global fish production, including capture and aquaculture, both for human consumption and ancillary purposes, is still growing worldwide, mainly due to the contribution of the aquaculture sector (Figure 1A) (FAO, 1996, 2002, 2004, 2010, 2012, 2016, 2018, 2020). In fact, 52% of the fish biomass produced for human consumption currently derives from aquaculture activities (FAO, 2020). Global fish production is dominated by China (35%), closely followed by the remainder of the Asian continent (34%), Americas (14%), Europe (10%), Africa (7%) and Oceania (1%) (FAO, 2020).

Given the obvious growth of the aquaculture industry and the emerging pressures it causes on human and environmental health, including the spread of bacterial diseases, this review covers alternative approaches to antibiotics and antimicrobials usage to suppress bacterial diseases in fish larvi- and aquaculture. Where appropriate, we place focus on studies of the model, cultured teleost fish gilthead seabream (*Sparus aurata*), an economically valuable reared species of relevance in Mediterranean countries. Our approach to pathogenicity in aquaculture emphasizes the opportunistic *Vibrio* species, highlighting environmental and public health concerns resulting from seafood vibriosis as well as human vibriosis acquired *via* seafood ingestion. In this context, the term vibriosis is herein defined as any sort of disease with clearly observable symptoms caused by *Vibrio* species on an animal host. PCR-based detection of virulence factors and mass spectrometry protocols used in the identification of *Vibrio* pathogens in fish and shellfish are thoroughly examined. Further emphasis is given to the existence of determinants of antibiotic resistance in *Vibrio* species present in commercial seafood products, given that they increase the risk of spread of antibiotic resistance genes from aquaculture to the consumer. New approaches for prophylaxis and treatment of vibriosis in fish relying on the management of pathobiomes and microbial communities in the aquaculture sector are then discussed, including the application of vaccines, bacteriophages, and probiotics to prevent bacterial disease proliferation. Particularly, we provide an overview of probiotics-based studies designed to supress *Vibrio* spp. across a broad range of host animals and aquaculture settings, portraying a solid body of work accumulated during the last 30 years which evidences great potential in the administration of probiotics for the control of vibriosis.

#### Aquaculture Production of Gilthead Seabream *(Sparus aurata)*

The marine perch-like fish *Sparus aurata* (*Linnaeus*, 1758), commonly known as gilthead seabream, is an economically valuable cultured species in southern European countries (Balebona et al., 1998b), ranking along with seabass as the most important fish species farmed in the Mediterranean zone (Firmino et al., 2019). World gilthead seabream aquaculture production, with regard to both quantity (tonnes) and value (thousand US$), has shown a consistent, continuous growth during the past three decades (Figures 1B,C).

Because of its plasticity and high amenability to rearing conditions, gilthead seabream can be cultured following extensive and semi-intensive methods in coastal ponds and lagoons. The extensive method relies partially on the species' natural migration and subsequent caught into fishing traps. Source juveniles obtained this way are, then, usually supplemented with additional juveniles reared in hatcheries by most of the modern stations employing the extensive method (FAO, 2021). A starting juvenile pool is, this way, seeded into a coastal lagoon, with juveniles (c. 45 DAH) weighting 2–3 g on average. Under this system, a juvenile achieves the first commercial size of 350 g in 20 months, with an average yield of 15–30 Kg/ha/yr and fish densities usually not exceeding 0.0025 kg/m3. Within semi-intensive rearing conditions, the increase of inputs derived from human activities (e.g., artificial feed and supplemental oxygen) results in a greater average production yield of 500–2,400 kg/ha/yr and higher fish densities of c. 1 kg/m^3^ (FAO, 2021).

**Figure 2:**
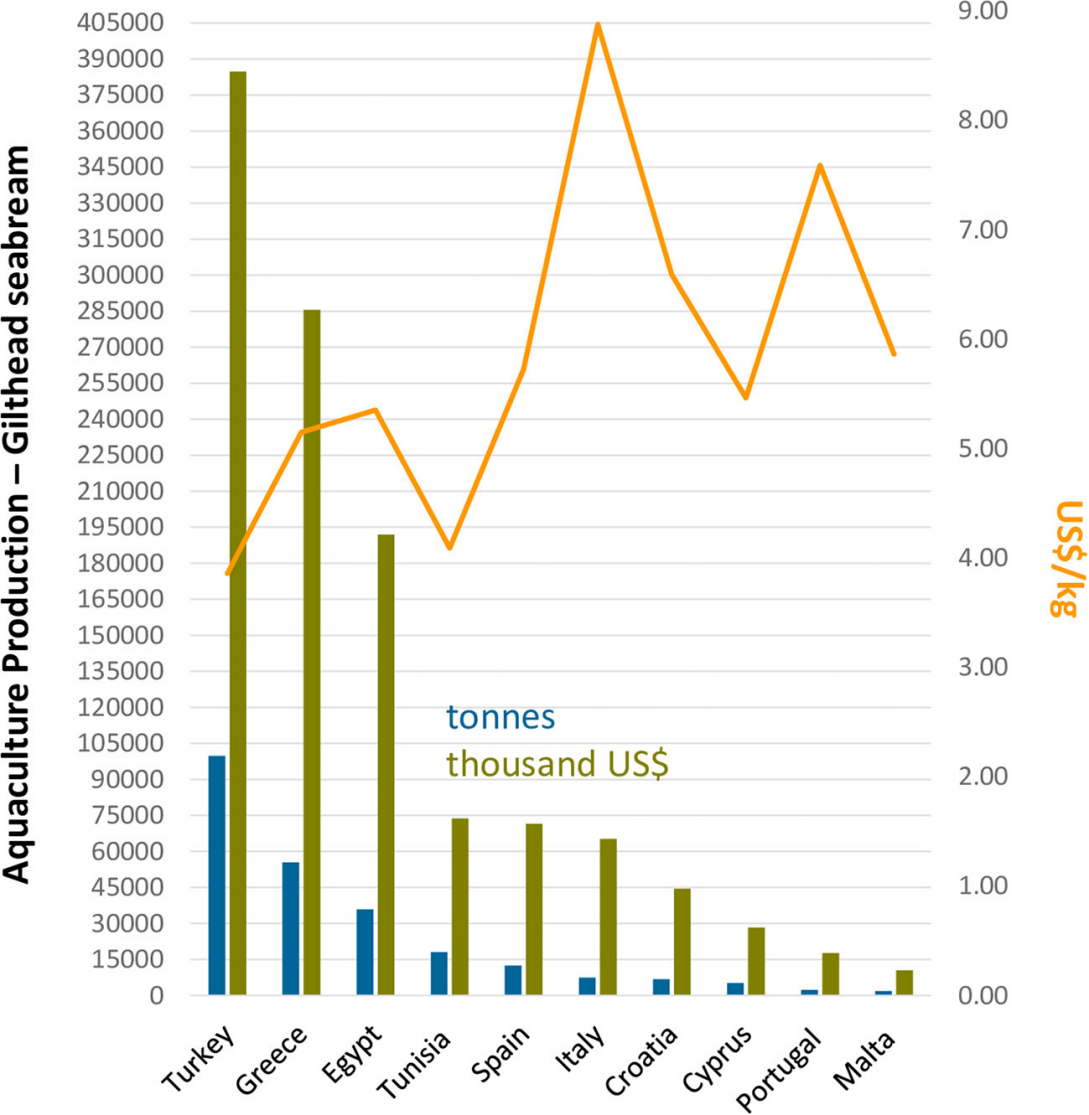
The top ten gilthead seabream producing countries in the Mediterranean zone. Colored bars represent quantity (tonnes) and commercial value (thousand US$/kg) of cultured gilthead seabream biomass produced for human consumption in 2019. The primary *Y*-axis represents quantity in tonnes, while the secondary axis represents the commercial value per kg of cultured gilthead seabream sold. Data collected from FAO, query online, http://www.fao.org/fishery/statistics/global-aquaculture-production/query/en.

Intensive rearing methods, in their turn, result in much higher yields in comparison with extensive and semi-intensive rearing methods. The densities of fish grown under this system, when raised in tanks receiving massive oxygen supply under optimal temperature conditions (18–26°C), are typically very high (15–45 kg/m^3^). In these circumstances, pre-fattened 5 g gilthead seabream may achieve the first commercial weight of 350 g in 1 year (FAO, 2021). Rearing of gilthead seabream in sea cages is a widely adopted methodology in the Mediterranean Sea, whereby reared fish densities may reach up to 10–15 kg/m3. Although intensive fish biomass production in sea cages is somewhat lower than that of land-based installations, the profits are much higher as there are no energy costs for pumping, aeration, or post-rearing water treatment (FAO, 2021). The main disadvantage is the absence of temperature control in sea cages and, consequently, the longer rearing period needed to reach the commercial size and to stock larger juveniles. Under this method, the larger, pre-fattened gilthead seabream (10 g) may take 1 year to reach the first commercial size of 350–400 g, while smaller seeding juveniles (5 g) achieve the same size in 16 months (FAO, 2021). Figure 2 lists the top ten gilthead seabream producing countries in the Mediterranean area. Interestingly, although not ranking among the three most producing countries in terms of quantity (tonnes/year), Italy (8,88 US$/kg), Portugal (7,59 US$/kg), Croatia (6,59 US$/kg), and Spain (5,73 US$/kg), in this order, were the countries presenting the highest commercial value of cultured gilthead seabream sold in the market.

### Aquaculture Microbiology

Aquaculture facilities constitute a high-density species environment where the use of live feed, the stress and the animals' physical proximity increase the propagation of parasites and diseases (Guidi et al., 2018; Sanches-Fernandes et al., 2021a). Therefore, monitoring quality, safety, and microbiological indicators, across all production stages, will play a decisive role in the development of future, sustainable and cost-effective aquaculture practices (FAO, 2020). One main problem to be overcome is the fact that most fish species display very low survival rates during larval rearing, which can be partially attributed to the spread of bacterial diseases (Snoussi et al., 2008; Sanches-Fernandes et al., 2021a). In fact, bacterial diseases are responsible for mass stock mortalities in fish farms throughout Mediterranean waters, independently of the reared species and host developmental stage (Bordas et al., 1996; Akayli and Timur, 2002; Kahla-Nakbi et al., 2006), resulting in a significant production bottleneck. In this context, it is not only essential to move beyond traditional microbiological assessments of food items using more efficient methodologies, in particular next generation DNA sequencing, to ensure effective monitoring of microorganisms in animal and plant-derived foods and tissues (Lorenzo et al., 2018). It is also important to advance our understanding of the roles played by beneficial microorganisms in aquaculture facilities to effectively steer these built ecosystems toward a more environmentally friendly state, whereby disease proliferation and pollution can be mitigated using natural resources (Vadstein et al., 2013; Borges et al., 2021). Borges et al. (2021) have recently reviewed the diversity and properties of potentially beneficial microbes that occur in aquaculture facilities, including a vast diversity of *Alphaproteobacteria* species belonging to the *Roseobacter* clade (e.g., *Phaeobacter inhibens*) which rank as promising probiotic candidates to control bacterial diseases in these settings (see more in section Microbial-Based Strategies to Prevent Vibrio Diseases in Aquaculture).

In intensive larval rearing of commercial fish species, live feed provision is still mostly required, usually including rotifers (*Brachionus* spp.) as the first feed item provided, followed by brine shrimp *Artemia* sp. at nauplii and metanauplii developmental stages, according to the mouth size of the growing fish larvae (Pousão-Ferreira, 2009). Although strains of the genera *Pseudomonas* (Skjermo and Vadstein, 1993; Rombaut et al., 2001), *Aeromonas* (Dhert et al., 2001), *Flavobacterium* (Skjermo and Vadstein, 1993; Dhert et al., 2001; Rombaut et al., 2001), *Marinomonas* and *Pseudoalteromonas* (Rombaut et al., 2001) have been quite commonly found in cultured rotifers, *Vibrio* species were the dominant bacteria associated with these animals according to early, cultivation-dependent studies (Verdonck et al., 1997). Altogether, the ingestion of rotifers and *Artemia* is a potential mechanism of transport of various pathogens into fish larvae. In addition, intake of pathogens from water by fish larvae is a concern, even within recirculation aquaculture systems (RAS), which are regarded as the safest in terms of disease control (Vadstein et al., 2013). Therefore, in larviculture facilities which need to use high densities of both rotifers and *Artemia* as live feed to fish larvae, the high load of organic matter present in water increases the risk of proliferation of opportunistic pathogenic bacteria to the developing fish host (Verdonck et al., 1997; Rombaut et al., 2001; Prol-García et al., 2010; Haché and Plante, 2011; Asok et al., 2012; Vadstein et al., 2013; Interaminense et al., 2014). Section *Vibrio* species and Vibriosis in Aquaculture provides an overview of *Vibrio* spp. already reported in association with fish live feed.

The pathogenic load in fish larviculture stations is believed to be determined by the complexity and diversity of the microbial communities occurring in the microhabitats that constitute these multifaceted, man-made ecosystems, including the rearing water itself, particulate organic materials deriving from animal excretions and dietary foods, the fish host, and the live feed (Califano et al., 2017). As such, host-microbe, microbe-microbe, and microbe-environment interactive forces that prevail in each rearing setting are thought to determine the final state of aquaculture microbiomes across a theoretical symbiome–pathobiome continuum. As our ability to catalog the diversity and function of host-associated microbiomes in a cultivation-independent manner increases, the molecular mechanisms underpinning host colonization, persistence and disease development by opportunistic microorganisms are predicted to be revealed at a fast pace. It is relevant that the functional attributes of pathobiomes and symbiomes of fish larvae, juveniles and live feed are uncovered, so that disease control in aquaculture can be implemented in a consistent manner (Borges et al., 2021). Notably, there is currently a demand for the development of faster, more precise, and accurate molecular methods (see section Identification of *Vibrio* Pathogens in Aquaculture), beyond common rRNA gene amplicon sequencing, to better identify the microorganisms present in the pathobiome, opening new avenues to understand pathogenic mechanisms in aquaculture (Vayssier-Taussat et al., 2014).

## *Vibrio* Species and Vibriosis in Aquaculture

*Vibrio* species are Gram-negative, asporogenous rods that are straight or curved, motile in aqueous environments usually by means of a single, polar flagellum (Kaysner et al., 2004). *Vibrio* spp. are mesophilic and chemoorganotrophic, possessing facultative fermentative metabolism (Kahla-Nakbi et al., 2007). They are ubiquitous inhabitants of aquatic environments including estuaries, marine coastal waters and sediments, and aquaculture settings (Balebona et al., 1998a; Thompson et al., 2004; Sarjito et al., 2009; Ringø, 2020). Except for *V. cholerae* and *V. mimicus* (Wong and Griffin, 2018), they are considered halophilic organisms (Wong and Griffin, 2018) commonly occurring at 30–35 ppt salinity although their aptitude to thrive in estuarine environments is also well-documented. The first *Vibrio* species described was *Vibrio cholerae*, in 1854, in the context of a study on cholera outbreaks in Florence (Thompson et al., 2004), but there are records of cholera-like diseases occurring in the times of Hippocrates (460–377 BC) (Blake, 1994). Currently, more than 130 species grouped in 14 clades in the *Vibrio* genus are recognized (Romalde et al., 2014; Huang et al., 2020), including commensal, mutualistic, and pathogenic species (Thompson et al., 2004).

The role of *Vibrio* spp. in marine organic carbon cycling (Romalde et al., 2014), particularly in coastal environments and marginal seas, has been underestimated (Zhang et al., 2018). *Vibrio* species are one of the best model marine heterotrophic bacterial groups, consuming several carbon compounds and growing with generation times as short as ~10 min (Zhang et al., 2018). They may represent about 60% of the total heterotrophic bacteria associated with aquatic organisms (Sonia and Lipton, 2012), being part of the normal microbiota of aquatic animals. *Vibrio* hosts are typically zooplankton, shellfish, crustaceans, benthic marine invertebrates such as sponges, corals and bryozoans, and fishes (Liu et al., 2016). Host-*Vibrio* relationships in nature may range from mutualistic through commensalistic to pathogenic (Liu et al., 2016). In general, *Vibrio* species proliferate well at warm temperatures, a condition that may favor their transition from commensal to pathogenic behavior. Environmental factors, including warming, have also been suggested to suppress fish immunity and increase their susceptibility to vibriosis (Haenen et al., 2014; El-Bouhy et al., 2016; El-Sayed et al., 2019).

In aquaculture, several *Vibrio* spp. are currently considered pathogens or opportunistic pathogens of reared finfish, shellfish, and shrimp (Liu et al., 2016). The most common *Vibrionaceae* spp. recorded in association with fish and shellfish diseases are *V. anguillarum, V. ordalii, V. vulnificus, V. alginolyticus* (Vera et al., 1991; Kahla-Nakbi et al., 2007; Korun and Timur, 2008), *V. parahaemolyticus* (Hamdan et al., 2016), *Aliivibrio* (formerly *Vibrio*; Urbanczyk et al., 2007) *salmonicida, V. harveyi* (Kahla-Nakbi et al., 2007; Korun and Timur, 2008), and *V. tubiashii* (Richards et al., 2014). Most of these species have been isolated both from reared and wild marine fish (Abdelaziz et al., 2017). Although *V. cholerae* is not referred to as a primary fish pathogen following Koch's postulates, it has been isolated from several freshwater and marine fish, which are considered a broad reservoir of *V. cholerae* strains that may cause infections in humans (Halpern and Izhaki, 2017). More recently, Devi et al. (2022) reported on a non-O1, non-O139 *V. cholerae* serotype (EMM1) capable of inducing high mortality in the freshwater species *Labeo rohita*, suggesting that *V. cholerae* strains other than the typical human pathogens shall be considered relevant aquatic pathogens as well. Vibriosis caused by the abovementioned species is the most common and devastating bacterial disease in fish larviculture and aquaculture, being a public health and economical concern affecting marine fishes, crustaceans, and bivalves worldwide (Balebona et al., 1998a; Sarjito et al., 2009; Ringø, 2020). The symptoms of vibriosis in fish are diverse, and include haemorrhagic septicaemias with extensive external skin lesions (haemorrhagic fins and ulcers), focal necrosis of some organs (liver, spleen, kidney), other tissue necrosis (Kahla-Nakbi et al., 2007), complete erosion of tail (Haldar et al., 2010), pale kidney, dark pigmentation, exophthalmic eyes, splenomegaly (Zorrilla et al., 2003), skeletal deformity (lordosis) (Abdel-Aziz et al., 2013), loss of appetite and lethargy (Korun and Timur, 2008).

Regarding the presence of *Vibrio* spp. in association with the live feed used for fish larviculture, several studies reported the isolation of the well-known causative agents of disease *Vibrio alginolyticus* (Yu et al., 1990), *V. anguillarum* (Dhert et al., 2001), *V. parahaemolyticus* (Balebona et al., 1998b) and *V. rotiferianus* (Gomez-Gil et al., 2003) from rotifers. The aquatic crustacean genus *Artemia* may also host *Vibrio* (Igarashi et al., 1989; Pousão-Ferreira, 2009), *Pseudomonas* (Igarashi et al., 1989) and *Aeromonas* (Interaminense et al., 2014) species. For instance, *V. alginolyticus* (Soto-Rodriguez et al., 2003; Interaminense et al., 2014), *V. parahaemolyticus* (Interaminense et al., 2014; Kumar et al., 2018), *V. anguillarum* (Campbell et al., 1993; Skjermo and Bergh, 2004), *V. harveyi* (Asok et al., 2012) and *V. hispanicus* (Gomez-Gil et al., 2004) have already been isolated from *Artemia*. Luminescent vibriosis (that is, vibriosis caused by luminescent *Vibrio* species) was as well-reported in *Artemia* and found to be caused mainly by *V. harveyi* and occasionally by *V. splendidus* (Soto-Rodriguez et al., 2003). Moreover, *V. campbellii*, frequently misidentified as *V. harveyi* in the past, was more recently found to be the etiological agent of luminescent vibriosis in shrimp hatcheries (Kumar et al., 2021).

## Vibriosis as a Worldwide Threat to Humans

Severe vibriosis in humans can be acquired by ingestion of contaminated water and raw or undercooked seafood (Wachsmuth et al., 1994; Finkelstein et al., 2002; Arab et al., 2020; Håkonsholm et al., 2020). Clinically, a few *Vibrio* species, despite their prevalently marine/estuarine origin, are able to elicit disease in humans. These include *V. cholerae, V. parahaemolyticus, V. vulnificus* (Wachsmuth et al., 1994; Finkelstein et al., 2002; Arab et al., 2020), *V. alginolyticus* (Gomathi et al., 2013; Citil et al., 2015), *V. metschnikovii* (Gomathi et al., 2013; Arab et al., 2020; Konechnyi et al., 2021), *V. mimicus* (Hernández-Robles et al., 2021), *V. cincinnatiensis* (Brayton et al., 1986), *V. fluvialis* (Ramamurthy et al., 2014; Kitaura et al., 2020), *V. furnissi* (Dalsgaard et al., 1997) and *V. harveyi* (Arab et al., 2020; Brehm et al., 2020). Well-documented symptoms of vibriosis in humans caused by *Vibrio* species which act as fish pathogens in aquaculture settings are highlighted below.

*Vibrio alginolyticus* had been frequently documented in early studies of gilthead seabream disease outbreaks in Mediterranean aquaculture (Balebona et al., 1998a). In humans, this bacterium was found to be associated with gastroenteritis in immunocompromised patients (Reina et al., 1995; Gomathi et al., 2013), causing extra-intestinal diseases (Gomez-Gil et al., 2003; Snoussi et al., 2008), wound infection, cellulitis, seawater-related otitis media (Abdel-Aziz et al., 2013; Gomathi et al., 2013), soft tissues and septicemia (Gomathi et al., 2013).

*Vibrio parahaemolyticus* is a well-known fish pathogen possessing a broad range of occurrence (Kumar et al., 2018). This bacterium was first recognized as a seafood borne pathogen to humans during an outbreak in 1950 in Osaka, Japan, involving 272 patients and causing the death of 20 people after the ingestion of Shirasu, a semi dried juvenile sardine (Aly et al., 2020). *V. parahaemolyticus* is the major food-borne pathogen worldwide (Bresee et al., 2002; Kawatsu et al., 2006), causing, after the ingestion of raw or undercooked seafood, acute dysentery and abdominal pain leading to diarrhea, nausea, vomiting, fever, chills, water-like stools, and an accentuated decrease of blood pressure leading to shock (Broberg et al., 2011; Siddique et al., 2021; Tan et al., 2021). In severe cases, patients become unconscious, with recurrent convulsions, becoming pale or cyanotic, eventually resulting in death. Antibiotic treatment and oral rehydration are the most common procedures to cure infections caused by *V. parahaemolyticus*. For individuals with critical physical or immunodeficiency diseases, the best practice to avoid severe illness is not to consume seafood at all (Wang et al., 2015). In the 21st century, increasing human disease outbreaks attributed to *V. parahaemolyticus* in Asia (Matsumoto et al., 2000), North America and Chile (Martinez-Urtaza et al., 2005), Europe namely France and Spain (Martinez-Urtaza et al., 2005; Quilici et al., 2005), Africa and Russia (Nair et al., 2007) have been described.

*Vibrio vulnificus* is highly pathogenic to humans (Snoussi et al., 2008). This bacterium causes epizootic outbreaks in seabream fish and can be transmitted to humans by ingestion, being a well-known cause of cellulitis and septicaemia in fishermen (Vinh et al., 2006). Besides, *V. vulnificus* is also able to infect the human host through an open cut or wound, in extreme cases resulting in necrotizing fasciitis, limb amputation and fatal septicaemia in susceptible individuals (Williams et al., 2014). The wound infections could start after the handling of infected fish and seafood, especially shellfish and after the practice of aquatic activities such as swimming (Hamdan et al., 2016; Baker-Austin and Oliver, 2018), being the consequences more severe when associated illnesses such as liver diseases, diabetes, and immune disorders are documented (Baker-Austin and Oliver, 2018). As usual among *Vibrio* spp., *V. vulnificus* possesses remarkable iron sequestration capabilities, meaning that the risk of infection is higher in humans with elevated iron levels (Wong and Griffin, 2018). More than 50% of primary septicaemia result in death within the first 72 h of hospitalization (Yun and Kim, 2018). Therefore, when there is a suspicion that the infection is caused by *V. vulnificus*, immediate and adequate antibiotic treatment and surgical interventions must be implemented. *V. vulnificus* is responsible for over 95% of deaths associated with seafood occurrences in the United States of America (Baker-Austin and Oliver, 2018). This is the highest fatality rate of any food-borne pathogen, which is in the range of category Biosafety Level 3 and 4 pathogens, namely anthrax, bubonic plague, Ebola, and Marburg fever (Baker-Austin and Oliver, 2018).

More recently, a few human infections caused by *V. harveyi* have as well been reported, underscoring the need of the public health sector to be aware of the possibility that wound infections caused by *Vibrio* species to humans may be becoming more likely to occur. With global warming, *Vibrio*-associated diseases will likely increase in the future (Brehm et al., 2020).

To avoid severe illness in humans caused by the ingestion of seafood contaminated with *Vibrio* species, thermal-based food processing such as low-temperature freezing (−18 or −24°C) or a 10 min high-temperature treatment (above 55°C) is common practice (Wang et al., 2015). Moreover, high-pressure processing and irradiation (using safety radioactive materials limits) are used to eliminate *V. parahaemolyticus* in oysters, keeping their original flavor (Wang et al., 2015). Notwithstanding the efficacy of hygiene measures employed in the preparation and processing of seafood for human consumption, devising novel, sustainable and green mechanisms of bacterial disease prevention in intensive fish farming holds promise in mitigating the impacts of the aquaculture industry on the environment and the risks posed to human health.

## Vibriosis Outbreaks in Farmed and Wild Gilthead Seabream

In 1997, the World Bank estimated that disease losses in aquaculture were worth US$3 billion per year, with *Vibrio* spp. having an important role in those losses (Laczka et al., 2014). Two decades later, estimates of disease losses duplicated (Stentiford et al., 2017). It has been suggested that all cultured marine fish around the world may, to varying degrees, host opportunistic vibrio species (Akayli and Timur, 2002), what does not necessarily imply that disease is always elicited nor that all vibrio species are pathogenic or will present pathogenic behavior. Yet multiple vibriosis outbreaks have been reported in several countries, infecting many fish species (Colorni et al., 1981; Akayli and Timur, 2002; Korun and Timur, 2008). In the case of the Mediterranean Sea, which is the primary habitat of gilthead seabream and a semi-closed water body, there is a limited rate of water exchange with open oceans. Aquatic pollution resulting from sewage, industrial effluents, crude oil refineries, and oil exploration affects the response of cultured fish to local environmental conditions (Guidetti et al., 2002). These factors also facilitate the invasion of bacterial pathogens (*Vibrio, Streptococcus, Aeromonas, Pseudomonas*) and parasites (nematodes, digeneans, acanthocephalans) into rearing systems. The ongoing chronic degradation of the Mediterranean Sea, thus, is considered to negatively impact the aquaculture industry in most of the North African coast (Eissa et al., 2017). For example, it has been suggested that the deterioration of water quality by sewage and agriculture discharges correlates with high prevalence of vibriosis in wild fish in the Mediterranean coast (Abdelaziz et al., 2017). Seawater exposed to higher anthropogenic pollution was found to display higher frequencies of *Vibrio* species, highlighting the importance of good manufacturing and hygiene practices to prevent and overcome fish vibriosis, even if innovative and “green” approaches were applied in industrial and domestic facilities (Abdelaziz et al., 2017; Arab et al., 2020).

The analysis of ten outbreaks involving *Vibrio* infections, affecting both cultured and wild gilthead seabream in the Mediterranean Sea (Table 1) revealed that *V. alginolyticus* was the *Vibrio* species most frequently isolated from gilthead seabream, followed by *V. harveyi, V. splendidus, V. anguillarum, V. parahaemolyticus*, and *V. tubiashii*. In these studies, *Vibrio* isolates were mainly recovered from seabream liver, spleen, and kidney, followed by external lesions and gills, but also from brain, eyes, gut, hepatopancreas, eroded tail and blood (Table 1). Interestingly, *V. ichthyoenteri-like* strains were isolated only from asymptomatic gilthead seabream individuals (Pujalte et al., 2003).

**Table 1:** Outbreaks caused by *Vibrio* infections in farmed gilthead seabream.

**Outbreak**	***Vibrio* species isolated**	**Isolated from**	**References**
**Spain (1990–1996)** Bacteriological survey 132 fish	*V. fischeri* (17.0%) *V. harveyi* (15.6%) *V. alginolyticus* (13.5%) *V. anguillarum* (12.8%) *V. splendidus* (10.6%) *V. nereis* (8.5%) *V. tubiashii* (5.0%) *V. campbellii* (4.3%) *V. aestuarianus* (1.4%) *Vibrio* spp. (11.4%)	Liver, spleen, kidney, other affected organs or tissues	Balebona et al., 1998b
**Spain (1997–2000)** 25 outbreaks 80 larvae 80 fingerlings (0.05–25 gr)	*V. alginolyticus* (21.4%) *V. harveyi* (13.6%) *V. fischeri* (6.8%) *V. splendidus* (6.8%) *V. anguillarum* (5.8%) *Vibrio* spp., 15 strains (15.5%)	Liver, spleen, kidney, external lesions	Zorrilla et al., 2003
**Turkey (1999–2000)** 15 outbreaks 60 fishes Juveniles (1–2 gr) Older fish (150 gr)	*Vibrio* spp.	Liver, spleen, kidney, blood, body surface lesions	Akayli and Timur, 2002
**Spain (2002)** Bacteriological survey 40 larvae (30 DAH)^a^ 40 larvae (60 DAH)^a^, ^b^ 547 fishes (average weight 21.6 gr)	*V. harveyi* *V. splendidus* *V. ichthyoenteri*-like *V. fischeri* *V. alginolyticus* *V. tubiashii* *V. pelagius* *V. mediterranei* *V. diazotrophicus* *Vibrio* spp.	Head kidney occasionally from the liver in small fish	Pujalte et al., 2003
**Tunisia (2002–2004)** seven outbreaks Larvae juveniles	*V. alginolyticus* (71.4%) *V. harveyi* (28.6%)	Liver, spleen, kidney, external lesions	Kahla-Nakbi et al., 2007
**Tunisia (2006)** juveniles (7 gr, 8 cm length) older fish (220 gr, 20 cm length)	*V. alginolyticus*	Juveniles: white nodular skin lesions Older fish: liver, spleen, kidney, gills	Snoussi et al., 2008
**Malta (2009)** one epizootic outbreak Juveniles (130 gr, 17.7 cm length) fingerlings hatchery	*V. harveyi*	Infected eye, eroded tail, gut, gills, hepatopancreas	Haldar et al., 2010
**Egypt (Feb 2013–Aug 2013)** 100 larvae (0.035–0.04 gr) 25 fingerlings (10–29.16 gr) 25 juveniles (83.77–190 gr)	*V. alginolyticus* *V. parahaemolyticus*	Liver, spleen, kidney, gills, brain, external lesions	Abdel-Aziz et al., 2013
**Egypt (2017–2018)** 200 farmed gilthead seabream commercial size	*V. parahaemolyticus*	Liver, spleen, kidney, gills	Aly et al., 2020
**Algeria (2017–2018)** No outbreak reported 280 farmed gilthead seabream 70 wild gilthead seabream^b^ commercial size (weighing at least 300 g)	*V. alginolyticus* *V. cholerae* *V. fluvialis*	Skin, gills, intestinal content	Arab et al., 2020

a *DAH, days after hatching*.

b *With no Vibrio species detected*.

According to the Spanish outbreak (1990–1996) study performed by Balebona et al. (1998b), the species *V. anguillarum, V. alginolyticus, V. harveyi*, and *V. splendidus* were considered highly virulent for gilthead seabream by intraperitoneal inoculation, based on mean lethal dose (LD_50_) values between 10^4^ and 10^6^ CFU per g body weight. In a further disease outbreak study, *V. alginolyticus* and *V. harveyi* were identified as virulent to gilthead seabream with LD_50_ values between 10^5^ and 10^6^ CFU per g body weight (Kahla-Nakbi et al., 2007). In that study, *V. alginolyticus and V. harveyi* were isolated from the skin mucus of gilthead seabream, and no inhibitory effects of the skin mucus collected from gilthead seabream against those isolates was found. In fact, those *Vibrio* isolates showed remarkable serum resistance and were also able to adhere to skin mucus and grow using it as a nutrient source, suggesting high host colonization ability to eventually become an important infection risk.

Intriguingly, the etiological agents of human seafood-borne infections *V. alginolyticus, V. cholera*, and *V. fluvialis* were found in tissues of farmed gilthead seabream showing no disease symptoms (Arab et al., 2020), supporting the notion of a growing presence of the causing agent of cholera, *V. cholerae*, in farmed fish for human consumption (Halpern and Izhaki, 2017; Arab et al., 2020). Indeed, higher incidence of human pathogenic *Vibrio* species in coastal marine waters has been considered to result from climate change effects on the composition of marine microbial communities (Vezzulli et al., 2016). In this context, it is worth noting that vibriosis in cultured gilthead seabream has already been found to be induced by several factors such as transport stress, sudden temperature changes, low oxygen levels in water and handling procedures (Akayli and Timur, 2002). We posit that the trends observed in this review regarding gilthead seabream-*Vibrio* interactions are most likely applicable to a range of economically valuable fish species.

The continuous study of marine and estuarine microbiomes in coastal areas is of utmost relevance for a better understanding of long-term microbial community changes in highly productive ecosystems in the face of climate change. Such databases can guide the identification of beneficial microbes that can be used to mitigate the proliferation of opportunistic pathogens in immunocompromised hosts in built and open environments at large. Gilthead seabream and seabass are the main farmed species in the Mediterranean basin, and vibriosis was recently reported as the most common bacterial disease affecting these species (Muniesa et al., 2020). Based on the current literature, we argue that the occurrence of vibriosis in humans—and consequently the threats to human health posed by *Vibrio* species thriving in aquaculture settings—may be of a larger magnitude than previously thought. The frequency of human infections caused by estuarine and marine *Vibrio* spp. is likely to increase as ever-expanding intensive farming and global climate change synergistically interact to favor the proliferation of opportunistic microorganisms in livestock production systems (Reverter et al., 2020).

## Identification of *Vibrio* Pathogens in Aquaculture

Species-level identification of members of the *Vibrio* genus, in an effective and standardized way, is necessary for a better bacteriological monitoring of farmed fish and the rearing environment within aquaculture facilities (Mustapha et al., 2013). As biochemical methods of identification often misidentify or are unsuccessful at the species level, molecular approaches must be implemented as common, accurate procedures for *Vibrio* species identification in seafood (Mustapha et al., 2013). However, it is important to note that, owing to the large genetic heterogeneity and fast diversification within *Vibrio* species, often 16S rRNA gene sequencing alone does not suffice for unequivocal identification of environmental strains at the species level. Bacterial species belonging to the *Vibrio* genus can differ in 16S rRNA gene nucleotide sequence from <1% up to 6% (Montieri et al., 2010). Particularly in the case of closely related species, the sequencing of a single marker gene may not be enough for precise taxon differentiation. For instance, *V. parahaemolyticus* and *V. alginolyticus* show quite similar biochemical properties (Mustapha et al., 2013) and are nearly identical with regards to 16S rRNA gene sequences (Montieri et al., 2010), prompting researchers to develop early DNA-based fingerprinting methods to discern between strains belonging to these species (Sadok et al., 2013). Indeed, *V. alginolyticus* was early designated *V. parahaemolyticus* biotype 2 (Aly et al., 2020), bearing testimony to the close phylogenetic relationship between these species.

Owing to the low discriminating power of highly conserved marker genes in distinguishing close *Vibrio* relatives, molecular identification based on species-specific markers and virulence genes have been considered adequate methods for species-level identification of *Vibrio* isolates (Mustapha et al., 2013). Because *Vibrio* spp. are symbiotic bacteria usually living in the intestine of aquatic species in a facultative way, genomic factors involved in the establishment of symbiosis and in the processes of host colonization and persistence may have evolved to confer adaptive advantage to species thriving in subtly different micro-niches. Several so-called “virulence factors”, such as enterotoxins, haemolysins, cytotoxins, proteases, lipases, phospholipases, siderophores, adhesive factors and/or haemagglutinins are produced by pathogenic species (Zhang and Austin, 2005). These traits allow *Vibrio* strains to adhere to the epithelial cells of fish juveniles, to break the first barrier of natural defense and to colonize all internal organs inducing vibriosis signs (Colorni et al., 1981; Paperna, 1984; Snoussi et al., 2008). It is important to note, however, that some of the abovementioned traits are common to several bacterial species and may likewise constitute adaptive features of mutualistic symbionts of fish (Borges et al., 2021). Overall, the use of genes coding for virulence or host-colonization factors as phylogenetic markers for the molecular detection of *Vibrio* species has gained increasing attention lately as nucleotide heterogeneities within such genes may reveal the adaptive behavior of different *Vibrio* species. A few PCR-based molecular identification systems of *Vibrio* species are listed in Table 2. These include protocols targeting genes coding for virulence factors which have been proved useful in discerning between closely related *Vibrio* species or in providing solid diagnosis of renowned pathogens, as reviewed more thoroughly in Supplementary File S1. For instance, several *V. alginolyticus* identification methods have been established based on the detection of hemolysin and collagenase encoding genes (Abdallah et al., 2011; Mustapha et al., 2013), and specific detection of *V. parahaemolyticus* and *V. alginolyticus* has been achieved through the exploration of nucleotide differences in genes encoding for the virulence regulatory proteins ToxR and ToxS (Abdallah et al., 2011; Aly et al., 2020). Also, a conserved virulence pathogenic island among *Vibrio* species has been exploited in the development of specific detection systems for the pathogen *V. vulnificus* (Table 2, see Supplementary File S1 for details).

**Table 2:** Target genes, gene functions, and oligonucleotide primer sequences used for specific detection and identification of *Vibrio* species.

**Target gene/** ** function**	**Target organism**	**Host samples**	**Primer**	**Oligonucleotide sequences (5^**′**^-3^**′**^)**	**Product size**	**References**
16SrRNA rRNA	*Vibrio* species in general	Fish, shellfish	63f 763r	F: CAGGCCTAACACATGCAAGTC R: GCATCTGAGTGTCAGTATCTGTCC	700 bp	Montieri et al., 2010 Abdelaziz et al., 2017
*cola* collagenase	*V. alginolyticus*	Fish, shellfish Seabass Gilthead seabream	VA-F VA-R	F: CGAGTACAGTCACTTGAAAGCC R: CACAACAGAACTCGCGTTACC	737 bp	Abdallah et al., 2011 Moustafa et al., 2015 Abdelaziz et al., 2017
*Tdh* thermostable direct hemolysin	*V. alginolyticus*	Fish and shellfish	tdh-F tdh-R	F: CCATCTGTCCCTTTTCCTGC R: CCAAATACATTTTACTTGG	373 bp	Mustapha et al., 2013
*trh* tdh-related hemolysin	*V. alginolyticus*	Fish and shellfish	trh-R2 trh-R6	F: GGCTCAAAATGGTTAAGCG R: CATTTCCGCTCTCATATGC	250 bp	Mustapha et al., 2013
*tox*R Regulatory virulence factor protein	*V. alginolyticus*	Seabass Gilthead seabream	toxR-F toxR-R	F: TTTGTTTGGCGTGAGCAAGGTTTT R: GGTTATTTTGTCCGCCAGTGG	595 bp	Kahla-Nakbi et al., 2009
*tox*S Regulatory virulence factor protein	*V. alginolyticus*	Seabass Gilthead seabream	toxS-F toxS-R	F: CCACTGGCGGACAAAATAACC R: AACAGTACCGTAGAACCGTGA	640 bp	Kahla-Nakbi et al., 2009
*vpi* Virulence pathogenicity island	*V. alginolyticus*	Shrimp Fish, seawater	vpi1 vpi2	F: GCAATTTAGGGGCGCGACGT R: CCGCTCTTTCTTGATCTGGTAG	680 bp	Kahla-Nakbi et al., 2009
*ami*B amidase	*V. anguillarum*	Marine flounder	van-ami8 van-ami417	F: ACAT CATCCATTTGTTAC R: CCTTATCACTATCCAAATTG	409 bp	Hong et al., 2007
*col*A collagenase	*V. parahaemolyticus*	Seawater Seabass Gilthead seabream	VP-F VP-R	F: GAAAGTTGAACATCATCAGCACGA R: GGTCAGAATCAAACGCCG	271 bp	Abdallah et al., 2011
*tox*R Regulatory virulence factor protein	*V. parahaemolyticus*	Fish, shellfish Gilthead seabream	ToxR-4 ToxR-7	F: GTCTTCTGACGCAATCGTTG R: ATACGAGTGGTTGCTGTCATG	368 bp	Abdelaziz et al., 2017 Aly et al., 2020
*Vvh*A *V. vulnificus* hemolysin	*V. vulnificus*	Fish, shellfish	vvhA up vvhA dn	F: CGCCGCTCACTGGGGCAGTGGCTG R: CCAGCCGTTAACCGAACCACCCGC	387 bp	Abdelaziz et al., 2017

It has been reasoned that the use of gene-targeted molecular tools may facilitate prevention of an outbreak as they allow the identification of the potential pathogens present even in asymptomatic fish (Altinok and Kurt, 2004). Yet it is presumably challenging to implement multiple gene amplicon sequencing methods in routine diagnostics for each different pathogen. In this regard, matrix-assisted laser desorption/ionization time-of-flight mass spectrometry (MALDI-TOF MS) is an alternative technique often used in the identification of *Vibrio* species that may show advantages over PCR-based detection of phylogenetic marker genes. Indeed, MALDI-TOF MS is not labor-intensive, does not require highly trained operators, and is suitable for the processing of many samples in an automated, rapid, and cost-effective way (Li et al., 2018; Mougin et al., 2020). However, for accurate identification of closely related *Vibrio* species using MALDI-TOF MS, database choice is crucial (as in the case of species identification using phylogenetic marker genes). For instance, Moussa et al. (2021) found that correct discrimination of isolates belonging to the species *V. tubiashii*/*V. europaeus* and *V. owensii*/ *V. jasicida*/*V. campbellii* could not be achieved using some of the commonly available databases for MALDI-TOF MS-based classification. However, successful identification of diverse *Vibrio* isolates was achieved by Mougin et al. (2020) through the combined use of the Luvibase and Bruker v.9.0.0.0 databases. Thus, to fully exploit the potential of MALDI-TOF MS in fast and accurate identification of *Vibrio* species in aquaculture facilities, continuous development of comprehensive databases that allow discrimination between closely related *Vibrio* species is fundamental.

In conclusion, the current tools for fast identification of *Vibrio* pathogens in aquaculture facilities, or cultured fish, usually rely on the use of toxin-encoding genes, or other alternative functional marker genes, in targeted, PCR-based approaches (Abdallah et al., 2009, 2011; Aly et al., 2020) as well as on mass spectrometry protocols which have been gaining momentum in recent years (Mougin et al., 2020; Moussa et al., 2021). The combination of highly specific molecular identification of *Vibrio* pathogens, either by means of gene-targeted or mass spectrometry approaches, and broad characterization of total microbial communities *via* high-throughput 16S rRNA gene sequencing, for instance, is likely to become an effective approach to accurately determine the presence of opportunistic/pathogenic bacteria in complex microbial communities inhabiting aquaculture facilities, which may include beneficial bacteria with the ability to supress the spread of pathogens present in the community. The steady development of well-curated databases in support of molecular diagnostic tools will play a decisive role in enabling (i) the identification of multiple pathogenic agents present in a sample including understudied organisms, such as the likely emerging pathogenic species *V. chagasii* (Sanches-Fernandes et al., 2021b) and *V. jasicida* (Sanches-Fernandes et al., 2021c); (ii) targeting the specific group of pathogenic agents typical of each facility in a straightforward manner (Stentiford, 2017). Given that each aquaculture facility is unique, with singular and distinct features (Stickney, 2016), these approaches shall be used in a complementary way and considered in a case-by-case manner.

## Antibiotic Resistance of *Vibrio* Species in Aquaculture Settings

Infections caused by drug-resistant pathogens are responsible for 700,000 annual deaths in aquaculture, estimated to reach 10 million deaths as of 2050 (O'Neill, 2015). Recent studies suggest that antibiotic resistant bacteria may not only emerge in the environment due to the use of antimicrobial agents but also due to the increase of local temperature (MacFadden et al., 2018; Reverter et al., 2020; Pepi and Focardi, 2021), since it can affect bacterial cell physiology and promote mutagenesis, allowing antibiotic resistance mutations to take place early (Pepi and Focardi, 2021). Accordingly, the occurrence of *Vibrio* outbreaks is commonly higher during spring and summer seasons (Aly et al., 2020). Nevertheless, *V. anguillarum* was referred to as the etiological agent of fish vibriosis in both warm and cold waters in aquaculture facilities (Lages et al., 2019). There is, however, evidence that *Vibrio*–associated diseases are increasing in a global manner because of climate change and human activities (Vezzulli et al., 2016). This highlights the urgent need for more effective actions to combat not only the indiscriminate use of antimicrobial agents but also global climate change and warming (Reverter et al., 2020; Pepi and Focardi, 2021). The development of multi-resistance traits among pathogenic *Vibrio* spp. has been reported steadily across several aquaculture stations worldwide (Scarano et al., 2014; Aly et al., 2020; Deng et al., 2020; Dutta et al., 2021; see Supplementary Table S1), and it may be reasonable to argue that this trend results from the synergistic effects of past and currently unsupervised antibiotics use and higher water temperatures.

The common practice and overuse of antibiotic administration for prophylactic reasons in aquaculture is an important factor to consider regarding the increase in transfer of antibiotic resistance genes to land animals and human pathogens (Costa et al., 2015). This is a global public health concern exacerbated by the fact that the increment in multiple antibiotic resistance is also observed in food-borne pathogens, opportunistic pathogens, and the commensal microbiota of animals for human consumption, resulting in antibiotic resistance in the human gastrointestinal tract (Nguyen et al., 2014). With regards to the most crucial players involved in horizontal gene transfer among *Vibrio* cells, along with plasmids, phages, transposons and integrons are also genomic islands and integrating conjugative elements (Rodríguez-Blanco et al., 2012; Costa et al., 2015). The inheritance of resistance traits is often acquired *via* conjugation of resistance plasmids (R-plasmids), which commonly contain genes encoding resistance to multiple antibiotics. R-plasmids have been reported for *Vibrio* capable of transferring drug resistance traits such as *V. alginolyticus* (Gomathi et al., 2013).

Resistance profiles of *Vibrio* species isolated from diseased gilthead seabream (including the most important *Vibrio* pathogens of fish and humans, such as *V. alginolyticus, V. harveyi, V. parahaemolyticus* and *V. vulnificus*) toward antibiotics frequently used in the aquaculture industry are summarized in Supplementary Table S1. This meta-analysis reveals that antibiotic resistance profiles may vary among strains of the same *Vibrio* species or across studies of the same species, which is the case of the data gathered for *Vibrio alginolyticus* and *V. harveyi*. Furthermore, we observed that most *Vibrio* species are sensitive to tetracycline, oxytetracycline, chloramphenicol, and florfenicol. All studies listed in Supplementary Table S1 were performed in gilthead seabream rearing facilities in the Mediterranean zone. Collectively, the data suggest a trend for increased antibiotic resistance among diverse *Vibrionaceae* species at the Mediterranean basin, possible to observe across time for species such as *V. aestuarius* (from 1998 to 2014), *V. alginolyticus* (from 1998 to 2014, where studies published in 2007 and 2008 were performed in the same region by the same research group), *A. fischeri* (from 1998 to 2003), *V. harveyi* (from 1998 to 2020), *V. parahaemolyticus* (from 2013 to 2020) and *V. splendidus* (from 1998 to 2003).

A more responsible and prudent use of antibiotics in the aquaculture sector is important as they are present in all production stages. In 2011, oxytetracycline was the antibiotic with the highest prescription for both prophylactic and therapeutic ends in aquaculture facilities, and also the one that is most of the times freely available (Bondad-Reantaso, 2018). There is currently no international uniformization regarding antibiotics usage approval, which are licensed by each country in accordance with their own legislation (Guidi et al., 2018). For an overview of antibiotics currently in use in aquaculture and existing policies among major producing countries, we refer the reader to the recent review by Lulijwa et al. (2020). We list some of the most frequently used antibiotics in the aquaculture sector and in the Mediterranean area in Supplementary Table S2, whereby those antibiotics approved for use in Norway, Italy, Brazil, and the United States are disclosed. We observed that *Vibrio* species are sensitive to several antibiotics licensed for use (Supplementary Table S1), including oxytetracycline and florfenicol. While this picture is congruent with the need of applying effective measures to deter vibriosis outbreaks, it simultaneously raises concerns regarding the development of broader multidrug resistance traits among *Vibrio* species. Perhaps as important as delineating which antimicrobials are permissible in what quantities and where, surveillance of the fate of antibiotics in the environment and seafood biomass is key to ensure adherence of farming stations to local/national policies. In this regard, it is worth noting that the concentration of permitted antibiotics in seafood biomass often exceeds maximum residual limits in most of the major producing countries (Lulijwa et al., 2020). This calls for an urgent up-scaling of surveillance capabilities for better traceability and follow-up of antibiotic use practices (Schar et al., 2020).

The fact that antibiotics have been usually applied for prophylactic, therapeutic, and metaphylactic purposes favors the loss of susceptibility among the target organisms, and hence an increasing trend of antibiotic resistance among *Vibrio* species is likely as suggested by the data present in this review. Although several antibiotics have been banned or subjected to strict regulations, particularly in industrialized countries, the legacy effects of their past and current indiscriminate use turn the development of multidrug resistance among bacterial pathogens an important and timely public health concern (Pepi and Focardi, 2021).

## Microbial-Based Strategies to Prevent *Vibrio* Diseases in Aquaculture

The negative impact of the over usage of antibiotics on farmed fish species and coastal environments worldwide urges the development of alternative methods to prevent disease proliferation and reduce ecosystem deterioration caused by emerging, multi-resistant opportunistic bacteria. The development of strategies that target bacterial pathogens based on the activation of the host's immune system (i.e., using vaccines), on biological interactions such as pathogen predation (i.e., phage therapy) and on competition/niche displacement among microbes or beneficial host-microbe interactions (e.g., using probiotics), are gaining increasing attention because they may offer a less hazardous alternative regarding the suppression of fish pathogens in aquaculture.

For an overview on the use of vaccines to prevent fish diseases, we refer the reader to the comprehensive reviews of Embregts and Forlenza (2016) and Ma et al. (2019), the latter on the promises and challenges of oral vaccine administration. In short, vaccination programmes are considered an efficient, pathogen-specific suppressive approach that is best employed for disease prevention among adult fish, especially when injection methods of antigen delivery are adopted (Embregts and Forlenza, 2016). Indeed, the implementation of efficient vaccination programmes in Norway during the nineties is nowadays considered a remarkable example of how alternative disease control methods can sharply reduce the use of antibiotics in intensive fish farming (Lulijwa et al., 2020). Presently, commercial *Vibrio* vaccines such as AquaVac™Vibromax™ (Wongtavatchai et al., 2010) and ALPHA JECT 3000 (PHARMAQ AS, Norway; see Torres-Corral et al., 2021 for an example of application) are available, which offer efficacy in vibriosis control in shrimp and finfish, respectively, under different administration methods, namely incubation of *Artemia nauplii* prior to shrimp feeding (AquaVac^TM^Vibromax^TM^; see Amatul-Samahah et al., 2020 for a review on vaccination of shrimp against vibriosis) and intraperitoneal injection of adult fish (AlphaJect 3000).

To improve fish wellbeing under vaccination programmes, oral administration methods using several modes of antigen encapsulation in delivery systems such as chitosan, alginates, and fish live feed such as *Artemia* and rotifers (bioencapsulation methods) have been attempted. However, improvements are still needed to ensure efficacy in antigen delivery in comparison with injection procedures. The main challenges of oral vaccine administration using encapsulation methods are to assure that vaccines reach the digestive tract of fish by ingestion, the maximum dosage allowed, which is dependent on the daily live feed intake, the time of exposure to be effective and the farmed fish tolerance to the vaccine (Embregts and Forlenza, 2016). The use of vaccines has moreover been considered not applicable to handle fish larvae and bivalves due to the lack of an adaptive immune system (Bentzon-Tilia et al., 2016), prompting researchers to consider alternative routes for disease prevention, such as the use of probiotics (see sub-section The Promise of Probiotics in Controlling Vibrio Diseases in Aquaculture).

Concerning phage therapy methods to prevent bacterial proliferation in aquaculture, the review by Richards (2014) covers pioneering studies on diverse bacteriophage-bacterial host systems and the efficacy of phage-based treatments to deter pathogens such as *Aeromonas samonicida, Edwardsiella tarda*, and *V. harveyi*, among others (Richards, 2014 and references therein). Like the vaccination approach, a key feature of phage therapy is its usual pathogen-specific nature, although the extent of specificity of the host-phage interaction may vary in case-dependent manner (Richards, 2014). In this regard, the use of phage mixtures has been considered a reasonable strategy to avoid the development of phage resistance by specific bacterial hosts while enabling the control of diverse pathogens (Richards, 2014). As for the use of vaccines and probiotics, phage dosage and delivery mode (immersion, oral *via* e.g., live feed ingestion, or injection) are crucial aspects for successful implementation of phage therapy (Richards, 2014; Soliman et al., 2019). To be cost-effective, phase dosage must be the lowest possible to induce bacterial infection with an associated, high phage replication rate (Soliman et al., 2019). Therefore, the use of lytic—instead of lysogenic—phages has been suggested as an imperative for the development of successful phage therapy methodologies (Richards, 2014). Since the ability to isolate and manipulate bacteriophages is strictly limited to the range of culturable bacterial hosts that can be captivated in the laboratory, and because the aquaculture pathobiome may include unculturable, or hard-to-culture, understudied bacteria, an intrinsic hurdle of the phage therapy approach relates with the development of novel methodologies leading to the control of bacterial populations for which no corresponding bacteriophages are known to date. Finally, large-scale application of phage therapy approaches in aquaculture shall be taken with caution, as concerns related with the environmental release of phages and its associated risks have been raised (Meaden and Koskella, 2013). The use of probiotics as a third, microbiome-based therapy approach to disease prevention in aquaculture is addressed below, as well as its likelihood to modulate aquaculture microbiomes toward a sustainable healthy state.

### The Promise of Probiotics in Controlling *Vibrio* Diseases in Aquaculture

Probiotics can be defined as live bacterial species able to survive and thrive in the acidic gastric environment whose activity leads to a beneficial effect on the health of the host by re-establishing or improving the gut microbiota, when administered in adequate amounts (Zhou et al., 2020; Moroni et al., 2021), although less stringent definitions have been proposed (see Borges et al., 2021). Probiotics are ideally inoffensive and promote host fitness. A mandatory feature of commercially successful probiotics is their viability during storage and on/in the animal host. Their application may follow reasonably standardized and easy-to-implement methodology if commercial formulations are deployed (Abareethan and Amsath, 2015). Probiotics may be administered to the fish host through several mechanisms, including inoculation of the rearing water, of formulated foods or of the live feed (Verschuere et al., 2000). The use of non-pathogenic biological agents as probiotics can be highly advantageous as they may act successfully as anti-bacterial, anti-viral and anti-fungal agents (Chauhan and Singh, 2019), thus presenting the potential to increase reared fish health and rearing water quality globally (Abareethan and Amsath, 2015). This attractive way to face, prevent and combat disease among reared fishes requires (host) species-specific studies to be made on the advantages of probiotics application, as symbiotic bacteria may act as pathogenic or probiotic depending on the aquatic host species. To properly address the molecular mechanisms of action elicited by probiotics on the immune system of the host, if any, further research is needed (Hai, 2015).

Probiotics-based therapies for disease control, when applied to farmed fish, should ideally consider the use of strains native to the host. This ensures higher probabilities of effective host colonization and persistence by the probiotics in use, at the operational rearing conditions, ultimately promoting nutrient acquisition by the host and a safer environment to the reared species, humans, and surrounding ecosystems (Wanka et al., 2018; Borges et al., 2021). Several modes of action have been reported for effective probiotics. These comprise the biosynthesis of inhibitory compounds that avoid pathogen proliferation, amelioration of the host immune system, competition with pathogens for adhesion sites in the gut or for essential nutrients, and even improvement of rearing water quality (Verschuere et al., 2000; Pérez-Sánchez et al., 2014). Probiotics were also reported as a source of nutrients, fatty acids, and vitamins to the fish host, and as possessing the capacity to enhance the digestibility of foods by the host organism through modulation of the fish gut microbiome (Pérez-Sánchez et al., 2014; Borges et al., 2021). Best practices to evaluate the potential of novel probiotic organisms usually involve the performance of *in vitro* antagonism tests, exposing pathogens to potential probiotic strains or to extracellular products synthesized by them in liquid and/or solid medium. To determine the ability of a probiotic strain to prevent disease and epizootic outbreaks, it is necessary that *in vivo* tests are performed (Verschuere et al., 2000; Pérez-Sánchez et al., 2014).

The information regarding the use of probiotic approaches in farmed gilthead seabream is still very scarce and even absent concerning larviculture facilities. Promising clues to follow to evaluate the potential of probiotics to prevent, or even treat, vibriosis in gilthead seabream larvi- and aquaculture facilities are suggested from the information gathered in Table 3, which broadens our scope to list studies showing empirical evidence of the efficacy of probiotics use to supress vibriosis across a wide range of host organisms. Several benefits were identified in shellfish aquaculture associated with the use of diverse probiotics, namely *Lactobacillus* spp., *Enterococcus* spp., *Bacillus* spp., *Aeromonas* spp., *Alteromonas* spp., *Arthrobacter* spp., *Bifidobacterium* spp., *Clostridium* spp., *Paenibacillus* spp., *Phaeobacter* spp., *Pseudoalteromonas* spp., *Pseudomonas* spp., *Rhodosporidium* spp., *Roseobacter* spp., *Streptomyces* spp. and even *Vibrio* spp. (Ringø, 2020, Table 3). The main benefits include fish growth promotion, improved digestive capacity, inhibition of adherence and colonization of pathogenic bacteria in the digestive tract, gut microbiota modulation, and the improvement of hematological parameters and the immune response (Ringø, 2020). Curiously, a previous study referred to many avirulent *V. alginolyticus* strains that could be used as probiotics (Akayli et al., 2008). *Vibrio alginolyticus* was also reported to possess probiotic effects against *Aeromonas salmonicida* (Hoseinifar et al., 2018). The use of *Bacillus cereus* isolated from the intestine of shrimps *Litopenaeus vannamei* is an example of successful re-colonization of the host intestine at the post-larval stage, probably due to competitive exclusion *via* the secretion of antimicrobial substances, especially resulting in effective suppression of *V. parahaemolyticus* and *V. harveyi*, justifying *Bacillus cereus* use as probiotic bacterium in shrimp larviculture (Vidal et al., 2018). This is an example of probiotic screening from natural host microbiomes that can be successfully applied across several aquaculture systems. Several other Gram-positive and Gram-negative probiotic bacteria showing suppressive features against *Vibrio* spp. have already been identified (Table 3).

**Table 3:** Overview of the effects of probiotics against pathogenic *Vibrio* species in farmed fish and shrimp.

**Probiotic strain**	**Pathogen**	**Fish species**	**Beneficial effects**	**Reference**
**Gram-positive bacteria**				
*Bacillus cereus*	*V. alginolyticus* IAL 1957	*Litopenaeus vannamei*	Significant decrease of pathogens by secretion of antimicrobial substances; competitive exclusion.	Vidal et al., 2018
*Bacillus licheniformis*	*V. alginolyticus*	*Macrobrachium rosenbergii*	Significant decrease in cumulative mortality; increased growth and immune response.	Kumar et al., 2013
*Bacillus subtilis E20*	*V. alginolyticus*	*Litopenaeus vannamei*	Immune modifications, such as increases in phenoloxidase activity, phagocytic activity, and clearance efficiency against vibriosis; increased survival.	Tseng et al., 2009; Wang et al., 2019
*Lactobacillus acidophilus 04*	*V. alginolyticus* SAC 15	*Penaeus monodon*	Effective pathogen inhibition; increased resistance and survival.	Natesan et al., 2012; Sivakumar et al., 2012
*Lactobacillus acidophilus NCIM 2285*	*V. alginolyticus*	*Penaeus indicus*	Effective pathogen inhibition; increased immune response and survival.	Ajitha et al., 2004
*Lactobacillus bulgaricus NCIM 2285 (2056)* *Lactobacillus bulgaricus NCIM 2285 (2057)*	*V. alginolyticus*	*Penaeus indicus*	Effective pathogen inhibition; increased immune response and survival.	Ajitha et al., 2004
*Lactobacillus fermentum* LW2	*V. alginolyticus*	*Litopenaeus vannamei*	Increased survival.	Wang et al., 2019
*Lactobacillus pentosus* BD6	*V. alginolyticus*	*Litopenaeus vannamei*	Increased survival.	Wang et al., 2019
*Lactobacillus plantarum*	*V. alginolyticus*	*Litopenaeus vannamei*	Immune modulation; increased resistance and survival.	Chiu et al., 2007; Ramírez et al., 2017
*Streptococcus cremoris* *NCIM 2285*	*V. alginolyticus*	*Penaeus indicus*	Effective pathogen inhibition; increased immune response and survival.	Ajitha et al., 2004
*Bacillus thuringiensis* *strain EA26.1*	*V. anguillarum*	*Litopenaeus vannamei*	Increased resistance to vibriosis.	Dou et al., 2016
*Carnobacterium divergens*	*V. anguillarum*	Atlantic cod	Decreased vibriosis.	Gildberg et al., 1997
*Clostridium butyricum CB2*	*V. anguillarum*	*Miichthys miiuy*	Increased phagocytic activity of leucocytes and therefore disease resistance to vibriosis.	Pan et al., 2008
*Clostridium butyricum* MIYAIRI	*V. anguillarum*	Rainbow trout	Increased disease resistance.	Sakai et al., 1995
*Enterococcus gallinarum* L1	*V. anguillarum* 975-1	Seabass	Decrease in mortality rates. Moderate protective effect; Extracellular substance production with antagonistic effect; Biding sites' competition on the intestinal mucus with a rate of exclusion of 66.2%.	Sorroza et al., 2013
*Kocuria* SM1	*V. anguillarum*	Rainbow trout	Decrease in mortality rates; stimulation of innate immune parameters.	Sharifuzzaman and Austin, 2010; Sharifuzzaman et al., 2011
*Lactococcus lactis subsp. lactis*	*V. anguillarum* ATCC 12486	*Litopenaeus vannamei*	Increased growth performance, digestive enzyme activity, disease resistance and survival.	Adel et al., 2017
*Pediococcus pentosaceus* 4012	*V. anguillarum*	Grouper	Significant decrease in cumulative mortality.	Huang et al., 2014
*Rhodococcus SM2*	*V. anguillarum*	Rainbow trout	Decrease in mortality rates.	Sharifuzzaman et al., 2011
*Vagococcus fluvialis*	*V. anguillarum* 975-1	Seabass	Increased survival rate.	Sorroza et al., 2012
*Bacillus sp. JL1*	*V. campbellii* *LMG 21363*	*Penaeus monodon*	Increased the survival, growth and robustness. Potential immunostimulatory strategy.	Laranja et al., 2014, 2017
*Bacillus* sp. NFMI-C	*V. campbellii BB120 (ATCC BAA-1116)*	*Macrobrachium rosenbergii*	Decreased quorum sensing-regulated luminescence of *V. campbellii*; Significantly higher survival.	Pande et al., 2015
*Lactobacillus pentosus* AS13	*V. campbellii*	*Litopenaeus vannamei*	Higher growth performance and digestive enzyme activities in the gut; Significantly lower mortality rate.	Zheng and Wang, 2017
*Bacillus aryabhattai* TBRC8450	*V. harveyi* 1562	*Litopenaeus vannamei*	Better shrimp innate immunity and antioxidant capacity; increased survival.	Tepaamorndech et al., 2019
*Bacillus cereus*	*V. harveyi*	*Penaeus monodon*	Potent growth promoter and immune enhancer.	NavinChandran et al., 2014
*Bacillus cereus*	*V. harveyi*	*Litopenaeus vannamei*	Increased survival.	Masitoh et al., 2016
*Bacillus cereus (DQ915582)*	*V. harveyi* MTCC 3438	*Penaeus monodon*	Increased resistance to vibriosis; Enhance survival.	Ravi et al., 2007
*Bacillus flexus* LD-1	*V. harveyi*	*Litopenaeus vannamei*	Increased growth, innate immune and digestive enzyme activities, stress tolerance, disease resistance.	Cai et al., 2019
*Bacillus licheniformis* LS-1	*V. harveyi*	*Litopenaeus vannamei*	Increased growth, innate immune and digestive enzyme activities, stress tolerance, disease resistance.	Cai et al., 2019
*Bacillus sp*. Mk22	*V. harveyi*	*Penaeus monodon*	Effective pathogen control.	Ashokkumar and Mayavu, 2014
*Bacillus P64*	*V. harveyi* (S2)	*Litopenaeus vannamei*	Significantly higher global immunity index.	Gullian et al., 2004
*Bacillus* S11	*V. harveyi* D311	*Penaeus monodon*	Shrimp appeared healthy and normal; competitive exclusion of pathogenic bacteria; 100% survival.	Rengpipat et al., 1998
*Bacillus* S11	*V. harveyi* D311 *V. harveyi* 1526	*Penaeus monodon*	Significantly higher survival; immune response stimulation, activation of cellular and humoral immune defenses.	Rengpipat et al., 2000
*Bacillus subtilis* L10 *Bacillus subtilis* G1	*V. harveyi* ATCC 14126	*Litopenaeus vannamei*	Higher immune response; improved growth performance and disease resistance.	Zokaeifar et al., 2012
*Bacillus subtilis* S12	*V. harveyi*	*Litopenaeus vannamei*	Significantly lower mortality; higher phagocytic rate and antibacterial activity. Effective immunopotentiator.	Liu et al., 2014
*Bacillus subtilis* P11	*V. harveyi* 639	*Penaeus monodon*	Increased immunity and survival.	Utiswannakul et al., 2011
*Bacillus subtilis* P11	*V. harveyi* 639	*Litopenaeus vannamei*	Increased disease resistance and survival.	Sapcharoen and Rengpipat, 2013
*Bacillus subtilis* BT23	*V. harveyi*	*Penaeus monodon*	Decrease in cumulative mortality.	Vaseeharan and Ramasamy, 2003
*Bacillus subtilis* S11	*V. harveyi* 639	*Litopenaeus vannamei*	Increased disease resistance and survival; Larger probiotic effect compared with *Bacillus subtilis* P11	Sapcharoen and Rengpipat, 2013
*Bacillus thuringiensis*	*V. harveyi*	*Litopenaeus vannamei*	Increased survival.	Masitoh et al., 2016
*Clostridium butyricum*	*V. harveyi*	*Macrobrachium rosenbergii*	Significantly higher digestive protease and amylase activities in the gastrointestinal tract; increased immune response.	Sumon et al., 2018
*Lactobacillus sp* *AMET1506*	*V. harveyi*	*Penaeus monodon* *Litopenaeus vannamei*	Increased resistance and survival.	Karthik et al., 2014, 2016
*Lactobacillus plantarum MRO3.12*	*V. harveyi*	*Litopenaeus vannamei*	Increased resistance and survival.	Vieira et al., 2010 Kongnum and Hongpattarakere, 2012 Shefat, 2018
*Enterococcus faecalis*	*V. harveyi*	*Macrobrachium rosenbergii*	Higher weight gain and digestive enzymes activities.	Khushi et al., 2022
*Enterococcus faecium MC13*	*V. harveyi*	*Penaeus monodon*	Effective pathogen inhibition; increased survival.	Swain et al., 2009
*Paenibacillus polymyxa (DQ915580)*	*V. harveyi* MTCC 3438	*Penaeus monodon*	Increased resistance to vibriosis and survival.	Ravi et al., 2007
*Paenibacillus spp. (EF012164)*	*V. harveyi* MTCC 3438	*Penaeus monodon*	Increased resistance to vibriosis and survival.	Ravi et al., 2007
*Streptococcus phocae PI80*	*V. harveyi*	*Penaeus monodon*	Effective pathogen inhibition; increased survival.	Swain et al., 2009
*Streptococcus phocae PI80*	*V. harveyi* MTCC 3435	*Cyprinus carpio*	Pathogen suppression.	Kanmani et al., 2010
*Streptococcus phocae PI80*	*V. harveyi* MTCC 3435	*Penaeus monodon*	Reduced shrimp mortality; shrimp survival rate of 100%.	Kanmani et al., 2010
*Streptomyces* strains CLS-39	*V. harveyi*	*Penaeus monodon*	Higher total length and wet weight.	Das et al., 2010
*Bacillus thuringiensis* G5-8-3T02	*V. mimicus*	*Penaeus monodon*	Higher disease resistance, weight and length gain.	Anyanwu and Ariole, 2019
*Carnobacteria inhibens*	*V. ordalii*	Rainbow trout	Reduced mortality	Robertson et al., 2000
*Arthrobacter XE-7*	*V. parahaemolyticus*	*Litopenaeus vannamei*	Higher immune response (total hemocyte counts, percentage phagocytosis, respiratory burst activity, and serum phenoloxidase activity); higher resistance to vibriosis.	Li et al., 2008
*Bacillus cereus*	*V. parahaemolyticus* ATCC 17802	*Litopenaeus vannamei*	Significant pathogen suppression through the secretion of antimicrobial substances; competitive exclusion.	Vidal et al., 2018
*Bacillus coagulans*	*V. parahaemolyticus*	*Penaeus monodon*	Immunomodulatory effect; Higher levels of superoxide dismutase (SOD) and catalase activity.	Raghu et al., 2016
*Bacillus coagulans* ATCC 7050	*V. parahaemolyticus*	*Litopenaeus vannamei*	Improved growth and intestinal morphology; diverse intestinal microbiota; higher immune response and resistance to vibriosis.	Amoah et al., 2019
*Bacillus firmus*	*V. parahaemolyticus*	*Penaeus monodon*	Immunomodulatory effect; higher levels of superoxide dismutase (SOD) and catalase activity.	Raghu et al., 2016
*Bacillus sp*. Mk22	*V. parahaemolyticus*	*Penaeus monodon*	Effective pathogen control; higher antioxidant enzyme activities.	Ashokkumar and Mayavu, 2014 Ashokkumar et al., 2016
*Bacillus subtilis UTM 126*	*V. parahaemolyticus* PS-017	*Litopenaeus vannamei*	Effectiveness at decreasing vibriosis.	Balcázar et al., 2007
*Bacillus subtilis* WB60	*V. parahaemolyticus* KCCM 11965	*Litopenaeus vannamei*	Improved growth, immunity, histology, gene expression, digestive enzyme activity; Increased disease resistance, while replacing antibiotics.	Won et al., 2020
*Clostridium butyricum* CBG01	*V. parahaemolyticus*	*Litopenaeus vannamei*	Improved growth performance, immunity capacity and resistance against vibriosis; Positive effect on the intestinal morphological structure.	Li et al., 2019
*Enterococcus faecium MC13*	*V. parahaemolyticus*	*Penaeus monodon*	Effective pathogen inhibition; increased survival.	Swain et al., 2009
*Lactococcus lactis* SGLAB02	*V. parahaemolyticus* (VP_AHPND_)	*Litopenaeus vannamei*	Immune system modulation; improved pathogen resistance.	Chomwong et al., 2018
*Lactobacillus bulgaricus E20*	*V. parahaemolyticus* PS-017	*Litopenaeus vannamei*	Better immune response in shrimp; higher survival rate and disease resistance.	Roomiani et al., 2018
*Lactobacillus pentosus HC-2*	*V. parahaemolyticus* E1	*Litopenaeus vannamei*	Improved immune responses, growth performance and disease resistance. Competitive exclusion of *V. parahaemolyticus* E1 in the intestine of shrimp.	Sha et al., 2016a,b
*Lactobacillus plantarum* SGLAB01	*V. parahaemolyticus* (VP_AHPND_)	*Litopenaeus vannamei*	Immune system modulations; improved pathogen resistance.	Chomwong et al., 2018
*Lactobacillus plantarum* T8	*V. parahaemolyticus* XN9 (AHPND)	*Litopenaeus vannamei*	Higher shrimp body length and weight; higher survival.	Nguyen et al., 2018
*Lactobacillus plantarum* T13	*V. parahaemolyticus* XN9 (AHPND)	*Litopenaeus vannamei*	Higher survival.	Nguyen et al., 2018
*Lactococcus lactis*	*V. parahaemolyticus* KCCM 11965	*Litopenaeus vannamei*	Improved growth, immunity, histology, gene expression, digestive enzyme activity; Improved disease resistance, while replacing antibiotics.	Won et al., 2020
*Paenibacillus polymyxa* ATCC 842	*V. parahaemolyticus*	*Litopenaeus vannamei*	Higher shrimp growth, serum and hepatopancreas immune and antioxidant activities; Improved digestive enzyme activities and intestinal morphology; gut microbiota modulation; higher survival.	Amoah et al., 2020
*Pediococcus pentosaceus*	*V. parahaemolyticus* KCCM 11965	*Litopenaeus vannamei*	Improved growth, immunity, histology, gene expression, digestive enzyme activity; Higher disease resistance, while replacing antibiotics.	Won et al., 2020
*Streptococcus phocae PI80*	*V. parahaemolyticus*	*Penaeus monodon*	Shrimp pathogen suppression.	Kanmani et al., 2010
*Streptococcus phocae PI80*	*V. parahaemolyticus*	*Cyprinus carpio*	Shrimp pathogen suppression.	Kanmani et al., 2010
*Streptomyces* strains N7	*V. parahaemolyticus*	*Litopenaeus vannamei*	Significantly higher survival rate.	García-Bernal et al., 2017
*Streptomyces* strains RL8	*V. parahaemolyticus*	*Litopenaeus vannamei*	Higher weight gain and survival rates.	García-Bernal et al., 2017
*Lactococcus lactis D1813*	*V. penaeicida*	*Marsupenaeus japonicus*	Improved pathogen resistance and survival; immunomodulatory activity.	Maeda et al., 2014
*Streptomyces* strains CLS-28	*V. proteolyticus*	*Penaeus monodon*	Higher total length and wet weight; higher survival.	Das et al., 2010
*Lactobacillus pentosus* AS13	*V. rotiferianus*	*Litopenaeus vannamei*	Improved growth performance and digestive enzyme activities in the gut; Significantly lower mortality rate.	Zheng and Wang, 2017
*Lactobacillus pentosus* AS13	*V. vulnificus*	*Litopenaeus vannamei*	Improved growth performance and digestive enzyme activities in the gut; Significantly lower mortality rate.	Zheng and Wang, 2017
**Gram-negative bacteria**				
*Pseudomonas fluorescens AH2*	*V. anguillarum*	Rainbow trout	Improved survival through 46% reduction in accumulated mortality.	Gram et al., 1999
*Phaeobacter gallaeciensis* *BS107 (DSM 17395)*	*V. anguillarum* NB10 (serotype O1)	Cod larvae	Mortality decreased by approximately 10%.	D'Alvise et al., 2012
*Roseobacter* sp. 27-4	*V. anguillarum* *90-11-287* (serotype O1)	Turbot	Controlled *V. anguillarum* infection.	Planas et al., 2006
*Roseobacter* sp. 27-4	*V. anguillarum 90-11-287* (serotype O1)	Sc*ophthalmus maximus*	Significant reduction in cumulative mortality.	Hjelm et al., 2004
*Shewanella putrefaciens* Pdp11	*V. anguillarum*	*Sparus aurata*	Lower mortalities.	Chabrillón et al., 2006
*Pseudomonas sp. W3*	*V. harveyi* PSU 2015	*Litopenaeus vannamei*	Improved survival, growth, and weigh gain likely through immunomodulatory effects.	Rattanachuay et al., 2007, 2011
*Vibrio hepatarius P62*	*V. harveyi* (S2)	*Litopenaeus vannamei*	Significantly higher global immunity index.	Gullian et al., 2004
*Pseudoalteromonas* NC201	*V. nigripulchritudo*	*Litopenaeus stylirostris*	Improved shrimp immune response expression; higher transcriptional activity of the gene coding for the antimicrobial peptide *Litsty* PEN3 in larvae; 2-fold lower cumulative mortality.	Pham et al., 2014; Sorieul et al., 2018
*Pseudoalteromonas* CDA22	*V. parahaemolyticus*	*Litopenaeus vannamei*	Higher resistance to vibriosis.	Wang et al., 2018
*Pseudoalteromonas* CDM8	*V. parahaemolyticus*	*Litopenaeus vannamei*	Higher resistance to vibriosis.	Wang et al., 2018
*Roseobacter gallaeciensis SLV03*	*V. parahaemolyticus* PS-017	*Litopenaeus vannamei*	Higher survival; effectiveness at decreasing vibriosis.	Balcázar et al., 2007
*Vibrio alginolyticus UTM 102*	*V. parahaemolyticus* PS-017	*Litopenaeus vannamei*	Higher final weight and survival; Effectiveness at decreasing vibriosis.	Balcázar et al., 2007
*Pseudomonas aeruginosa* PsDAHP1	*V. parahaemolyticus* DAHV2 (GFP-VpDAHV2)	*Zebrafish*	Fish through biofilm formation inhibition and improved defense mechanisms.	Vinoj et al., 2015
*Pseudomonas aestumarina SLV22*	*V. parahaemolyticus* PS-017	*Litopenaeus vannamei*	Higher survival and final weight. Effectiveness at decreasing vibriosis.	Balcázar et al., 2007
*Aeromonas media A199*	*V. tubiashii*	*Crassostrea gigas*	Improved resistance to vibriosis.	Gibson et al., 1998

Antibiotic-producing bacterial probiotics such as *V. hepatarius* P62, *Pseudomonas* sp., *Lactobacillus* sp., *Bacillus* P64, along with yeast probiotics applied in shellfish and fish aquaculture have been usually selected from their natural environment (Cedeño and Rodríguez, 2006). The probiotic activity of *Saccharomyces cerevisiae* P13 against *V. alginolyticus* was demonstrated through the significant enhancement of survival rates of Pacific white shrimp *Litopenaeus vannamei* (Wang et al., 2019). *Bacillus pumilus* H2 could be very useful as an anti-*Vibrio* probiotic as it was shown to inhibit 29 different *Vibrio* strains (Gao et al., 2017). The anti-*Vibrio* compound was found to be amicoumacin, whose activity against *Vibrio* pathogens is based on the disruption of cell membranes, resulting in cell lysis. However, the minimum inhibitory concentration (MIC, expressed in μg/ml) of the purified anti-*Vibrio* compound amicoumacin A, isolated from *Bacillus pumilus* H2, was found to vary considerably depending on the *Vibrio* species/strain, from 0.5 μg/ml for *V. vulnificus* CZ-A2 and *V. harveyi* PH4 to 64 ug/ml for *V. alginolyticus* CGMCC 1.1607 and *V. parahaemolyticus* CGMCC 1.2164 (Gao et al., 2017).

Probiotic species able to inhibit diverse *Vibrio* strains or species in *in situ* experiments are scarcely documented. Future research should shed light on the potential use of *Bacillus pumilus* as a deterrent of multiple opportunistic species in aquaculture facilities. Tropodithietic acid (TDA) produced by *Phaeobacter* spp. can protect live feed, namely rotifers and *Artemia*, as well as turbot larvae and cod larvae against pathogenic *Vibrio* species (Rasmussen et al., 2018) such as *V. anguillarum* (D'Alvise et al., 2012). It was also found that the probiotic bacterium *Phaeobacter inhibens* strain S4Sm inhibited the growth of *V. tubiashii* and *V. anguillarum* in cultured oysters (Zhao et al., 2016). Furthermore, *Phaeobacter inhibens* antagonized *V. anguillarum* in cultures of copepod and in the copepod live feed *Rhodomonas salina* (Rasmussen et al., 2018), emerging as another candidate probiotic species with the ability to suppress multiple *Vibrio* species.

A multitude of readily culturable bacteria possessing potential probiotic features are currently available and well-described. These can be explored for the implementation of novel and effective methodologies of pathogen suppression, for example involving the development of multi-species probiotic inoculants or of smart delivery systems (e.g., using alginates) that may enhance the host colonization ability of probiotics. However, despite all the promising advances mentioned above, only three probiotic strains - the gut microbiota stabilizers *Pediococcus acidilactici* CNCM MA 18/5M and *Pediococcus acidilactici* CNCM I-4622 (bacteria), and the digestibility enhancer yeast *Komagataella pastoris* DSM 23036 - are authorized by the European Union to be used as live organisms in aquaculture facilities [European Commission 2021, Reg (EC) No 1831/2003]. In face of the acute challenges posed by increasing disease incidence in the aquaculture sector, and of the manifold possibilities of microbiome-based disease control revealed in the last three decades, it is reasonable to argue that time is ripe for advancing new legislation that is on par with current scientific knowledge regarding the use of environmentally safer, bio-based methodologies to deter bacterial diseases in this sector.

## Concluding Remarks

Microorganisms are major contributors to nutrient cycling and functioning within aquaculture facilities yet a fraction of the total microbiome thriving in these man-made is also responsible for disease and mortality affecting live feed, fish larvae, fish, and shellfish. The extent to which solutions to the pathogenicity problem within aquaculture facilities may be found in the naturally occurring aquaculture microbiome is a matter of current scientific debate. The effective implementation of protocols relying on the use of vaccines, phage therapy and probiotics holds promise in deterring pathogen proliferation in intensive fish farming. For instance, a wealth of Gram-positive and Gram-negative bacteria showing remarkable capacities to supress *Vibrio* pathogens or mitigate vibriosis symptoms in farmed fish and shellfish have been identified in the past 30 years. Nevertheless, to move beyond the proof-of-concept stage, such protocols still face technical and legal challenges that prevent their wide-range applicability at the production scale. Better understanding of microbiomes thriving in “healthy” and “diseased” hosts and aquaculture systems is key to instruct researchers in the pursuit of techniques leading to efficient microbiome manipulation and engineering toward safer rearing systems, eventually decreasing the need of using antibiotics and hazardous chemicals to control bacterial diseases in aquaculture.

The increasing pollution of coastal ecosystems caused by sewage and industrial effluent inputs, including oils, fertilizers, and heavy metals, may also result in a negative physiological response of reared fish, favoring the invasion of bacterial pathogens and parasites in rearing systems exposed to such pollutants. Therefore, proper environmental monitoring and ecosystem conservation are fundamental to prevent bacterial disease incidence in aquaculture. Aquaculture facilities may be in fact “hotspots” for gene transfer, as they contain dense and highly diverse bacterial communities whose structure and taxonomic composition result from the combination of current and past use of antibiotics, probiotics, prebiotics as well as other kinds of treatments or methods. In this context, it is important to discern the coding potential present in the mobile gene pool of *Vibrio* species and assess the intra- and interspecific transferability of these genes, as this bears implications to our understanding to the roles of *Vibrio* species as disease-causing agents and of the potential switch from commensal to pathogenic behavior based on processes of gene gain and loss in the *Vibrio*-associated plasmidome. Indeed, Bruto et al. (2017) revealed that pathogenicity of *Vibrio crassostreae* toward the cultured oyster species *Crassostrea gigas* is mediated by the acquisition of a large mobilizable plasmid. It is reasonable to argue that such processes mediate virulence of *Vibrio* pathogens of fish and may be promoted by high host densities in intensive rearing conditions.

Vibriosis is one of the most important diseases causing high mortality rates in the aquaculture industry. According to the meta-analysis discussed in this review, species such as *V. alginolyticus* and *V. harveyi* are among the main responsible for epizootic disease outbreaks causing economic losses in this sector, affecting several fish species, including the production of reared gilthead seabream in the Mediterranean zone. As the frequency of antibiotic- and multidrug-resistance *Vibrio* spp. is growing, constant surveillance and monitoring of antibiotic resistance and pollution must be assured to avoid the development of multi-resistant strains which may pose threats to both ecosystem and human health. Although certain *Vibrio* species from diseased farmed gilthead seabream were found to be sensitive to tetracycline, oxytetracycline, chloramphenicol and florfenicol, the development of alternative, cost-effective and sustainable pathogen suppression methods in aquaculture is encouraged from an environmental and a human health standpoint. Particularly worrisome is the current rise of human infections caused by environmental and seafood-associated *Vibrio* species, apparently influenced by climate-change drivers of microbial community assembly in coastal ecosystems, including intensive seafood farming systems.

The major goal of aquaculture production is supplying food for human consumption. Following several decades of heavy use of antimicrobial drugs and antibiotics to boost intensive fish rearing, current prophylaxis approaches that contribute to a more health-oriented management of aquaculture systems are being increasingly recommended to prevent or suppress epizootic disease outbreaks. They involve the use of less dangerous methods such as vaccines, immunostimulants and probiotics/microbiome therapy. This way, it is believed that healthier food for human consumption may be produced and bacterial resistance to antibiotics may be prevented or alleviated, thus reducing the transfer of acquired antibiotic resistance traits to human pathogens *via* mobile genetic elements.

## Author Contributions

GMMS-F, IS-C, and RC conceived and designed the study. GMMS-F performed the literature research, prepared figures, and tables. GMMS-F and RC interpreted the data and wrote the first manuscript draft. All authors read and revised the first manuscript draft and approved the final manuscript.

## Funding

This study was supported by FCT—Fundação para a Ciência e a Tecnologia, I.P., through the research grants PTDC/MAR/112792/2009 and PTDC/BIA-MIC/31996/2017 and by the European Regional Development Fund (ERDF, Project # 031996, operational code ALG-01-0145-FEDER-031966) through the regional operational programs of Lisbon and Algarve, Portugal. This study was also financed by FCT in the scope of the projects UIDB/04565/2020 and UIDP/04565/2020 of the Research Unit Institute for Bioengineering and Biosciences—iBB, and the project LA/P/0140/2020 of the Associate Laboratory i4HB - Institute for Health and Bioeconomy.

## Conflict of Interest

The authors declare that the research was conducted in the absence of any commercial or financial relationships that could be construed as a potential conflict of interest.

## Publisher's Note

All claims expressed in this article are solely those of the authors and do not necessarily represent those of their affiliated organizations, or those of the publisher, the editors and the reviewers. Any product that may be evaluated in this article, or claim that may be made by its manufacturer, is not guaranteed or endorsed by the publisher.
