# Supplementary material for: Vibriosis Outbreaks in Aquaculture: Addressing Environmental and Public Health Concerns and Preventive Therapies Using Gilthead Seabream Farming as a Model System
Source: Front Microbiol. 2022 Jul 11;13:904815. doi: 10.3389/fmicb.2022.904815 (PMC9309886; doi:10.3389/fmicb.2022.904815)
Supplement: Supplementary file 1 [file Data_Sheet_1.pdf]

Supplementary File S1

Vibriosis outbreaks in aquaculture: addressing environmental and public health concerns and preventive therapies using gilthead seabream farming as a model system

Gracinda M.M. Sanches-Fernandes[1,2,3], Isabel Sá-Correia[1,2,3], Rodrigo Costa[1,2,3,4*]

[1]Institute for Bioengineering and Biosciences (iBB), Instituto Superior Técnico, Universidade de Lisboa, Lisbon, Portugal

[2]Department of Bioengineering, Instituto Superior Técnico, Universidade de Lisboa, Lisbon, Portugal

[3]Associate Laboratory i4HB—Institute for Health and Bioeconomy at Instituto Superior Técnico (IST), Universidade de Lisboa, Lisbon, Portugal

[4]Centre of Marine Sciences (CCMAR), University of Algarve, Portugal

*** Correspondence:**
Rodrigo Costa
rodrigoscosta@tecnico.ulisboa.pt

Keywords: Biological Control, Fish larviculture, Fish microbiome, Host-microbe interactions, Probiotics, *Vibrio*

Detailed Information

Identification of *Vibrio* Pathogens in Aquaculture
Here we provide more specific information on molecular-based studies used in the identification of *Vibrio* species, highlighted in Table 2 (Main Article).

The species-specific *pR72H* DNA sequence marker (of unknown function) was found to be highly conserved in *V. parahaemolyticus* strains, being used for species-specific detection by PCR amplification (Robert-Pillot *et al*., 2002). Species-specific primers targeting the *pR72H* gene were also used to identify *V. parahaemolyticus* in farmed gilthead seabream (Aly *et al*., 2020). *V. anguillarum* has as well been identified in *Oreochromis niloticus* fish by PCR using a specific primer set targeting the *V. anguillarum* alternative virulence marker *amiB* gene, encoding the peptidoglycan hydrolase N-acetylmuramoyl-L-alanine amidase, involved in the separation of daughter cells after cell division (Hong *et al*., 2007; Frans *et al*., 2013; Asran *et al*., 2020). Hemolysins are the most widely produced toxins among pathogenic *Vibrio* spp., playing various roles in the infection process, for instance, by acting on erythrocyte membranes leading to cell lysis and the release of iron-binding proteins such as haemoglobin, transferrin and lactoferrin. Virulence is therefore closely associated with the expression of thermostable direct hemolysin (TDH) and thermostable direct hemolysin -

related hemolysin (TRH), encoded by *tdh* and *trh* genes, respectively (Siddique *et al*., 2021; Tan *et al*., 2021). Molecular research based on virulence genes has been often applied for the identification of *V. alginolyticus* isolates in seafood, by searching the presence of the virulence genes *tdh* and *trh*, coding for the two hemolysins, TDH and TRH, respectively (Mustapha *et al*., 2013). In addition, the species-specific primer targeting the *V. alginolyticus* virulence gene *colA*, coding for the extracellular enzyme collagenase, was used in the identification of *V. alginolyticus* isolates from diseased farmed gilthead seabream (Moustafa *et al*., 2015) and wild moribund and freshly dead fish (Abdelaziz *et al*., 2017). Previous studies (Abdallah *et al*., 2009; Abdallah *et al*., 2011) found *V. cholerae* virulence genes *toxR*, *toxS* and the Virulence Pathogenicity Island (VPI) in the genome of *V. alginolyticus* strains isolated from diseased, farmed gilthead seabream. The membrane-localized regulatory protein ToxR, containing a cytoplasmic DNA-binding-transcriptional activation domain, a transmembrane domain, and a periplasmic domain (Montieri *et al*., 2010), has a crucial role in modulating bacterial persistence and virulence (Zhang *et al*., 2018). The ToxS regulatory protein, also located in the inner membrane, acts as mediator of ToxR function in the *V. cholerae* virulence regulon, called the ToxR regulon (Xu *et al*., 2010). In fact, the transmembrane transcription factor ToxR is activated and protected from degradation by its integral membrane periplasmic binding partner ToxS (Midgett *et al*., 2020). The Virulence Pathogenicity Island (VPI) was found to be an essential virulence gene cluster in epidemic *V. cholerae* (Rajanna *et al*., 2003), with pivotal roles in toxin production and disease development (Kumar *et al*., 2020). Although *toxR* and *toxS* genes are well conserved in the *Vibrionaceae* family (Midgett *et al*., 2020), a wide divergence in the region between transcriptional activation and transmembrane domains, known as “tether” region, has been previously reported (Montieri *et al*., 2010). In the case of *V. parahaemolyticus* and *V. alginolyticus*, there are only 61.7% identical nucleotides within the partial *toxR* gene sequence. Thus, the *toxR* gene is a useful molecular marker for phylogenetic analyses of *V. parahaemolyticus* and *V. alginolyticus* strains, given its clearly higher resolution in differentiating these species in comparison with the 16S rRNA gene (Montieri *et al*., 2010). In fact, fast methodologies purely based on the length of the *toxR* and *toxS* genes and of the *vpi* gene cluster have been successfully developed to identify *V. alginolyticus* strains via PCR (Abdallah *et al*., 2009; Abdallah *et al*., 2011). Likewise, identification of *V. parahaemolyticus* strains can be achieved via PCR amplification of the virulence *toxR* gene (Aly *et al*., 2020). Finally, isolates from diseased farmed fish were identified as *V. vulnificus* by targeting the virulence gene *vvhA*, which encodes the *V. vulnificus* hemolysin that contributes to bacterial invasion from the intestine into the bloodstream and other organs (Abdelaziz *et al*., 2017; Yuan *et al*., 2020).

Antibiotic Resistance of *Vibrio* species in Aquaculture Settings

Table S1 Antibiotic resistance profiles of *Vibrionaceae* species (and strains within species) isolated from diseased farmed gilthead seabream, towards several antibiotics commonly used in aquaculture.

Vibrio spp. isolates (Reference)	PCN	AMP	AMX	VAN	CTX	AMK	GEN	STR	TOB	KAN	TET	OTC	ERY	CHL	FLO	NAL	OXA	FLU	ENR	CIP	NIT	NOV	CEF	SDI	TMP	FZL	CLI	CAZ	LIN
V. aestuarianus (Balebona, 1998b)	0	0	0	n.d	n.d	100.	100.	100	100	100	0	0	100	0	n.d	0	0	0	n.d	n.d	0	n.d	0	n.d	0	0	0	n.d	0
V. aestuarianus (Scarano, 2014)	n.d	50.0	50.0	n.d	n.d	n.d	0	50.0	n.d	0	0	0	100	0	50.0	n.d	50.0	50.0	n.d	n.d	n.d	n.d	50.0	100	50.0	n.d	n.d	n.d	n.d
V. alginolyticus (Balebona, 1998b)	100	100	0	n.d	n.d	84.2	52.6	100	64.8	84.2	47.4	36.8	89.5	0	n.d	47.4	0	0	n.d	n.d	0	n.d	0	n.d	0	0	0	n.d	n.d
V. alginolyticus (Zorrilla, 2003)	n.d	100	100	n.d	n.d	n.d	n.d	n.d	n.d	n.d	0	0	n.d	n.d	n.d	n.d	0	4.5	n.d	n.d	n.d	n.d	n.d	n.d	9.1	n.d	n.d	n.d	n.d
V. alginolyticus (Kahla-Nakbi, 2007)	n.d	R	n.d	R	S	n.d	n.d	n.d	n.d	I	n.d	S	S	S	n.d	n.d	S	S	n.d	n.d	I	n.d	n.d	n.d	S	S	R	n.d	n.d
V. alginolyticus (Snoussi, 2008)	n.d	100	n.d	n.d	70.4	n.d	88.9	n.d	n.d	n.d	92.6	n.d	88.9	29.6	n.d	63.0	n.d	n.d	n.d	85.2	n.d	n.d	n.d	n.d	n.d	n.d	n.d	74.1	n.d
V. alginolyticus (Abdel-Aziz, 2013)	n.d	R	R	n.d	n.d	n.d	n.d	n.d	n.d	n.d	n.d	n.d	n.d	S	n.d	S	S	n.d	n.d	S	n.d	n.d	n.d	n.d	n.d	n.d	n.d	n.d	R
V. alginolyticus (Scarano, 2014)	n.d	80.0	82.0	n.d	n.d	n.d	2.0	20.0	n.d	4.0	0	2.0	36.0	0	0	n.d	16.0	20.0	n.d	n.d	n.d	n.d	56.0	74.0	16.0	n.d	n.d	n.d	n.d
V. anguillarum (Balebona, 1998b)	100	94.4	0	n.d	n.d	100	61.1	100	38.9	100	38.9	33.3	55.5	0	n.d	22.2	0	0	n.d	n.d	0	n.d	0	n.d	0	0	0	n.d	0
V. anguillarum (Zorrilla, 2003)	n.d	100	100	n.d	n.d	n.d	n.d	n.d	n.d	n.d	0	0	n.d	n.d	n.d	n.d	0	0	n.d	n.d	n.d	n.d	n.d	n.d	0	n.d	n.d	n.d	n.d
V. anguillarum (Canak, 2018)	n.d	R	n.d	n.d	n.d	n.d	n.d	R	n.d	R	n.d	I	R	n.d	S	n.d	n.d	I	I	I	n.d	n.d	n.d	S	n.d	n.d	n.d	n.d	n.d
V. campbellii (Balebona, 1998b)	100	100	0	n.d	n.d	0	0	100	0	0	0	0	100	0	0	0	0	0	n.d	n.d	0	n.d	0	R	0	0	0	n.d	0

V. casei (Scarano, 2014)	n.d	0	0	n.d	n.d	n.d	0	0	n.d	0	0	0	100	0	0	n.d	0	0	n.d	n.d	n.d	n.d	66.7	100	0	n.d	n.d	n.d	n.d
V. communis/owensii (Scarano, 2014)	n.d	100	100	n.d	n.d	n.d	0	0	n.d	0	0	0	0	0	0	n.d	0	0	n.d	n.d	n.d	n.d	0	100	0	n.d	n.d	n.d	n.d
V. diabolicus (Scarano, 2014)	n.d	81.8	90.9	n.d	n.d	n.d	9.1	0	n.d	0	0	0	27.3	0	9.1	n.d	0	0	n.d	n.d	n.d	n.d	81.8	90.9	9.1	n.d	n.d	n.d	n.d
V. fischeri (Balebona, 1998b)*	0	4.2	0	n.d	n.d	41.7	8.3	100	4.2	58.3	0	0	58.3	0	n.d	0	0	0	n.d	n.d	0	n.d	0	n.d	0	0	0	n.d	0
V. fischeri (Zorrilla, 2003)*	n.d	100	100	n.d	n.d	n.d	n.d	n.d	n.d	n.d	0	0	n.d	n.d	n.d	n.d	0	0	n.d	n.d	n.d	n.d	n.d	n.d	0	n.d	n.d	n.d	n.d
V. gigantis (Scarano, 2014)	n.d	100	100	n.d	n.d	n.d	100	100	n.d	0	0	0	100	0	0	n.d	0	0	n.d	n.d	n.d	n.d	100	100	100	n.d	n.d	n.d	n.d
V. harveyi (Balebona, 1998b)	100	100	0	n.d	n.d	50.0	40.9	0	45.4	63.6	0	0	100	0	n.d	0	0	0	n.d	n.d	0	n.d	0	n.d	0	0	0	n.d	0
V. harveyi (Zorrilla, 2003)	n.d	100	100	n.d	n.d	n.d	n.d	n.d	n.d	n.d	0	7.1	n.d	n.d	n.d	n.d	0	0	n.d	n.d	n.d	n.d	n.d	n.d	0	n.d	n.d	n.d	n.d
V. harveyi (Kahla-Nakbi 2007)	n.d	R	n.d	R	S	n.d	n.d	n.d	n.d	I	n.d	S	I	S	n.d	n.d	I	S	n.d	n.d	S	n.d	n.d	n.d	S	S	R	n.d	n.d
V. harveyi (Scarano, 2014)	n.d	87.3	87.3	n.d	n.d	n.d	1.8	14.5	n.d	1.8	1.8	1.8	80.0	1.8	5.5	n.d	3.6	9.1	n.d	n.d	n.d	n.d	14.5	76.4	27.3	n.d	n.d	n.d	n.d
V. harveyi (Canak, 2018)	n.d	R	n.d	n.d	n.d	n.d	n.d	R	n.d	R	n.d	I	I	n.d	S	n.d	n.d	I	I	I	n.d	n.d	n.d	n.d	I	n.d	n.d	n.d	n.d
V. harveyi/owensii (Scarano, 2014)	n.d	0	0	n.d	n.d	n.d	0	0	n.d	0	0	0	100	0	0	n.d	50.0	50.0	n.d	n.d	n.d	n.d	100	100	100	n.d	n.d	n.d	n.d
V. litoralis (Scarano, 2014)	n.d	0	50.0	n.d	n.d	n.d	0	0	n.d	0	0	0	100	0	0	n.d	0	0	n.d	n.d	n.d	n.d	0	50.0	0	n.d	n.d	n.d	n.d
*V. logei** (Canak, 2018)*	n.d	R	n.d	n.d	n.d	n.d	n.d	R	n.d	R	n.d	I	R	n.d	S	n.d	n.d	S	I	I	n.d	n.d	n.d	0	n.d	n.d	n.d	n.d	n.d
V. mediterranei (Scarano, 2014)	n.d	0	0	n.d	n.d	n.d	0	0	n.d	0	0	0	0	0	0	n.d	0	0	n.d	n.d	n.d	n.d	0	0	0	n.d	n.d	n.d	n.d

V. mytili (Scarano, 2014)	n.d	11.1	11.1	n.d	n.d	n.d	0	0	n.d	0	11.1	11.1	55.6	0	0	n.d	0	0	n.d	n.d	n.d	n.d	0	88.9	0	n.d	n.d	n.d	n.d
V. nereis (Balebona, 1998b)	0	0	0	n.d	n.d	83.3	0	100	66.7	91.7	0	0	100	0	n.d	0	0	0	n.d	n.d	0	n.d	0	n.d	0	0	0	n.d	0
V. ordalii/anguillarum (Scarano, 2014)	n.d	0	0	n.d	n.d	n.d	0	0	n.d	0	0	0	33.3	0	0	n.d	0	0	n.d	n.d	n.d	n.d	33.3	100	0	n.d	n.d	n.d	n.d
V. orientalis (Scarano, 2014)	n.d	0	0	n.d	n.d	n.d	0	0	n.d	50.0	0	0	100	50.0	50.0	n.d	50.0	50.0	n.d	n.d	n.d	n.d	0	100	100	n.d	n.d	n.d	n.d
V. orientalis/hepatarius (Scarano, 2014)	n.d	100	100	n.d	n.d	n.d	0	0	n.d	0	0	0	0	0	100	n.d	0	100	n.d	n.d	n.d	n.d	100	100	100	n.d	n.d	n.d	n.d
V. parahaemolyticus (Abdel-Aziz, 2013)	n.d	R	R	n.d	n.d	n.d	n.d	n.d	n.d	n.d	n.d	n.d	n.d	S	n.d	S	S	n.d	n.d	S	n.d	n.d	n.d	n.d	n.d	n.d	n.d	n.d	R
V. parahaemolyticus (Scarano, 2014)	n.d	25.0	0	n.d	n.d	n.d	0	25.0	n.d	0	0	0	25	0	0	n.d	0	0	n.d	n.d	n.d	n.d	25.0	100	0	n.d	n.d	n.d	n.d
V. parahaemolyticus (Aly, 2020)	n.d	90.6	n.d	n.d	n.d	n.d	73.4	n.d	n.d	n.d	n.d	17.2	n.d	n.d	n.d	n.d	n.d	n.d	n.d	29.7	n.d	0	n.d	n.d	0	n.d	n.d	n.d	n.d
V. scophthalmi (Canak, 2018)	n.d	I	n.d	n.d	n.d	n.d	n.d	R	n.d	I	n.d	S	R	n.d	S	n.d	n.d	S	S	S	n.d	n.d	n.d	S	n.d	n.d	n.d	n.d	n.d
V. splendidus (Balebona, 1998b)	93.3	93.3	0	n.d	n.d	6.7	66.7	93.3	93.3	0	0	0	73.3	26.7	n.d	20.0	0	0	n.d	n.d	0	n.d	0	n.d	0	0	0	n.d	0
V. splendidus (Zorrilla, 2003)	n.d	14.3	57.1	n.d	n.d	n.d	n.d	n.d	n.d	n.d	14.3	14.3	n.d	n.d	n.d	n.d	0	14.3	n.d	n.d	n.d	n.d	n.d	n.d	0	n.d	n.d	n.d	n.d
V. tubiashii (Balebona, 1998b)	0	0	0	n.d	n.d	85.7	71.4	85.7	71.4	85.7	0	0	100	0	n.d	20.0	0	0	n.d	n.d	0	n.d	0	n.d	0	0	0	n.d	0
V. tasmaniensis (Scarano, 2014)	n.d	100	100	n.d	n.d	n.d	0	50.0	n.d	0	0	0	100	0	100	n.d	0	0	n.d	n.d	n.d	n.d	100	100	100	n.d	n.d	n.d	n.d
V. vulnificus (Scarano, 2014)	n.d	0	0	n.d	n.d	n.d	0	0	n.d	0	0	0	0	0	0	n.d	100	100	n.d	n.d	n.d	n.d	0	100	0	n.d	n.d	n.d	n.d

PCN (penicillin), AMP (ampicillin), AMX (amoxicillin), VAN (vancomycin), CTX (cefotaxime), AMK (amikacin), GEN (gentamicin), STR (streptomycin), TOB (tobramycin), KAN (kanamycin), TET (tetracycline), OTC (oxytetracycline), ERY (erythromycin), CHL (chloramphenicol), FLO (florfenicol), NAL (nalidixic acid), OXA (oxolinic acid), FLU (flumequine), ENR (enrofloxacin), CIP (ciprofloxacin), NIT (nitrofurantoin), NOV (novobiocin), CEF (cephalothin), SDI (sulfadiazine), TMP (trimethoprim/sulfamethoxazole), FZL (furazolidone), CLI (clindamycin), CAZ (ceftazidime), LIN (lincomycin). Cells filled in red: resistant (resistance percentages from 50% to 100% or R). Cells filled in yellow: intermediately sensitive (resistance percentages from 11% to 49% or I). Cells filled in green: sensitive (resistance percentages from 0% to 10% or S). n.d.: not determined. Resistance percentage values were registered every time they were present in the original studies. Resistance percentages were calculated as the quotient between the number of strains isolated in gilthead seabream showing resistance against the antibiotic tested and the total number of strains tested with that antibiotic. The percentages reported by Snoussi *et al*. (2008) were recalculated as they included strains isolated from rearing water.

* *Aliivibrio* (formerly *Vibrio*) *fischeri* (Urbanczyk *et al*., 2007), ** *Aliivibrio* (formerly *Vibrio*) *logei* (Urbanczyk *et al*., 2007).

Table S2 – Antibiotics commonly used in Mediterranean aquaculture.

Antibiotic Class	Commonly used antibiotics*	References
Tetracyclines	oxytetracycline [(i)(ii)(iii)(iv)]	Rodgers *et al.*, 2009; Scarano *et al.*, 2014; Bondad-Reantaso, 2018; Guidi *et al.*, 2018; FDA, 2022; Lulijwa *et al.*, 2020
	chlortetracycline [(i)]	Rodgers *et al.*, 2009; Scarano *et al.*, 2014; Bondad-Reantaso, 2018
	doxycycline	Bondad-Reantaso, 2018
Quinolones	oxolinic acid [(i)(iv)]	Rodgers *et al.*, 2009; Bondad-Reantaso, 2018; Guidi *et al.*, 2018; Lulijwa *et al.*, 2020
	flumequine [(i)(iv)]	Rodgers *et al.*, 2009; Scarano *et al.*, 2014; Bondad-Reantaso, 2018; Guidi *et al.*, 2018; Lulijwa *et al.*, 2020
	enrofloxacin	Bondad-Reantaso, 2018; Guidi *et al.*, 2018
	sarafloxacin [(i)]	Rodgers *et al.*, 2009; Scarano *et al.*, 2014; Bondad-Reantaso, 2018; Guidi *et al.*, 2018
Phenicols	florfenicol [(i)(ii)(iii)(iv)]	Rodgers *et al.*, 2009; Bondad-Reantaso, 2018; Guidi *et al.*, 2018; FDA, 2022; Lulijwa *et al.*, 2020
	chloramphenicol	Bondad-Reantaso, 2018
	thiamphenicol	Bondad-Reantaso, 2018
Antifolates	trimethoprim-sulfamethoxazole [(i)]	Rodgers *et al.*, 2009; Bondad-Reantaso, 2018
	trimethoprim-sulfadiazine [(i)(iv)]	Scarano *et al.*, 2014; Lulijwa *et al.*, 2020
	ormetoprim-sulfadimethozine [(iii)]	FDA, 2022
Diaminopyrimidine	Trimethoprim [(i)]	Bondad-Reantaso, 2018
(antifolates)	ormetoprim	Guidi *et al.*, 2018
Sulfonamides (antifolates)	sulfadimethoxine	Bondad-Reantaso, 2018; Guidi *et al.*, 2018
β-lactams	amoxicillin [(i)]	Rodgers *et al.*, 2009; Scarano *et al.*, 2014; Bondad-Reantaso, 2018; Guidi *et al.*, 2018
Macrolides	erythromycin	Bondad-Reantaso, 2018; Guidi *et al.*, 2018
	josamycin	Bondad-Reantaso, 2018
	neomycin (for G+ve bacteria)	Bondad-Reantaso, 2018
Nitrofurans	nitrofurantoin	Bondad-Reantaso, 2018

*Antibiotics approved for aquaculture in [(i)] Italy (Scarano *et al.*, 2014), [(ii)] Brazil (Guidi *et al.*, 2018), [(iii)] United States (FDA, 2022; content current as of: 03/18/2022, https://www.fda.gov/animal-veterinary/aquaculture/approved-aquaculture-drugs) and (iv) Norway (Lulijwa *et al.*, 2020)

References

Abdallah, F. B., Bakhrouf, A., Ayed, A., and Kallel, H. (2009). Alterations of outer membrane proteins and virulence genes expression in gamma-irradiated *Vibrio parahaemolyticus* and *Vibrio alginolyticus*. *Foodborne Pathog. Dis*. 6, 1171-1176. doi:10.1089/fpd.2009.0331.

Abdallah, F. B., Ellafi, A., Lagha, R., Kallel, H., and Bakhrouf, A. (2011). Virulence gene expression, proteins secreted and morphological alterations of *Vibrio parahaemolyticus* and *Vibrio alginolyticus* in response to long-term starvation in seawater. *African J. Microbiol. Res.* 5, 792–801. doi:10.5897/ajmr10.653.

Abdel-Aziz, M., Eissa, A. E., Hanna, M., and Okada, M. A. (2013). Identifying some pathogenic *Vibrio*/*Photobacterium* species during mass mortalities of cultured gilthead seabream (*Sparus aurata*) and European seabass (*Dicentrarchus labrax*) from some Egyptian coastal provinces. *Int. J. Vet. Sci. Med.* 1, 87–95. doi:10.1016/j.ijvsm.2013.10.004.

Abdelaziz, M., Ibrahem, M. D., Ibrahim, M. A., Abu-Elala, N. M., and Abdel-moneam, D. A. (2017). Monitoring of different vibrio species affecting marine fishes in Lake Qarun and Gulf of Suez: phenotypic and molecular characterization. *Egypt. J. Aquat. Res.* 43, 141–146. doi:10.1016/j.ejar.2017.06.002.

Aly, S. M., Eisa, A. A., and Elbanna, N. I. (2020). Characterization of *vibrio parahaemolyticus* infection in gilthead seabream (*Sparus aurata*) cultured in Egypt. *Egypt. J. Aquat. Biol. Fish.* 24, 553–571. doi:10.21608/EJABF.2020.76562.Asran, E., Khalil, S., Awad, Y., and Hussein, M. (2020). Molecular characterization of *Vibrio* species isolated from fish using PCR technique. *Alexandria J. Vet. Sci.* 67, 9. doi:10.5455/ajvs.117363.

Balebona, M. C., Zorrilla, I., Moriñigo, M. A., and Borrego, J. J. (1998b). Survey of bacterial pathologies affecting farmed gilt-head sea bream (*Sparus aurata* L.) in southwestern Spain from 1990 to 1996. *Aquaculture* 166, 19–35. doi:10.1016/S0044-8486(98)00282-8.

Bondad-Reantaso, M. G. (2018). Country level implementation: FAO experience in Aquaculture, FAO Session 4: Responsible and prudent use of veterinary antimicrobials: practical tools and experiences. 2nd OIE Global Conference on Antimicrobial Resistance and Prudent Use of Antimicrobial Agents in Animals, Putting Standards into Practice. Marrakesh, Morocco. https://www.oie.int/amr2018/wp-content/uploads/2018/11/S4_3_Reantoso.pdf.

Canak, O., and Akayli, T. (2018). Bacteria recovered from cultured gilt-head seabream (*Sparus aurata*) and their antimicrobial susceptibilities. *Eur. J. Biol.* 77, 11–17. doi:10.26650/eurojbiol.2018.346175.

FDA (2022). Approved Aquaculture Drugs. U.S. Food & Drug Administration. Content current as of: 03/18/2022. https://www.fda.gov/animal-veterinary/aquaculture/approved-aquaculture-drugs.

Frans, I., Dierckens, K., Crauwels, S., Van Assche, A., Leisner, J., Larsen, M. H., et al. (2013). Does Virulence Assessment of *Vibrio anguillarum* Using Sea Bass (*Dicentrarchus labrax*) Larvae Correspond with Genotypic and Phenotypic Characterization? *PLoS One* 8, 2–10. doi:10.1371/journal.pone.0070477.

Guidi, L. R., Santos, F. A., Ribeiro, A. C. S. R., Fernandes, C., Silva, L. H. M., and Gloria, M. B. A. (2018). Quinolones and tetracyclines in aquaculture fish by a simple and rapid LC-MS/MS method. *Food Chem.* 245, 1232–1238. doi:10.1016/j.foodchem.2017.11.094.

Hong, G. E., Kim, D. G., Bae, J. Y., Ahn, S. H., Bai, S. C., and Kong, I. S. (2007). Species-specific PCR detection of the fish pathogen, *Vibrio anguillarum*, using the *amiB* gene, which encodes N-acetylmuramoyl-L-alanine amidase. *FEMS Microbiol. Lett.* 269, 201–206. doi:10.1111/j.1574-6968.2006.00618.x.

Kahla-Nakbi, A. Ben, Besbes, A., Bakhrouf, A., and Alcaide, E. (2007). Characterisation and virulence properties of *Vibrio* isolates from diseased gilthead sea bream (*Sparus aurata*) cultured in Tunisia. *Bull. Eur. Assoc. Fish Pathol.* 27, 90–99.

Kumar, A., Das, B., and Kumar, N. (2020). *Vibrio* Pathogenicity Island-1: The Master Determinant of Cholera Pathogenesis. Front. Cell. Infect. Microbiol. 10, 1–12. doi:10.3389/fcimb.2020.561296.

Lulijwa, R., Rupia, E. J., and Alfaro, A. C. (2020), Antibiotic use in aquaculture, policies and regulation, health and environmental risks: a review of the top 15 major producers. *Rev. Aquacult*. 12, 640-663. https://doi.org/10.1111/raq.12344

Midgett, C. R., Swindell, R. A., Pellegrini, M., and Jon Kull, F. (2020). A disulfide constrains the ToxR periplasmic domain structure, altering its interactions with ToxS and bile-salts. *Sci. Rep.* 10, 1–11. doi:10.1038/s41598-020-66050-5.

Montieri, S., Suffredini, E., Ciccozzi, M., and Croci, L. (2010). Phylogenetic and evolutionary analysis of *Vibrio parahaemolyticus* and *Vibrio alginolyticus* isolates based on *toxR* gene sequence. *New Microbiol.* 33, 359–372.Mustapha, S., Mustapha, E., and Nozha, C. (2013). *Vibrio alginolyticus*: an emerging pathogen of foodborne diseases. *Int. J. Sci. Technol* 2, 302–309.

Rajanna, C., Wang, J., Zhang, D., Xu, Z., Ali, A., Hou, Y. M., et al. (2003). The *Vibrio* Pathogenicity Island of Epidemic *Vibrio cholerae* Forms Precise Extrachromosomal Circular Excision Products. *J. Bacteriol.* 185, 6893–6901. doi:10.1128/JB.185.23.6893-6901.2003.

Robert-Pillot, A., Guenole, A., and Fournier, J. M. (2002). Usefulness of R72H PCR assay for differentiation between *Vibrio parahaemolyticus* and *Vibrio alginolyticus* species: Validation by DNA-DNA hybridization. *FEMS Microbiol. Lett.* 215, 1–6. doi:10.1016/S0378-1097(02)00884-4.

Rodgers, C. J., and Furones, M. D. (2009). Antimicrobial agents in aquaculture: practice, needs and issues. The use of veterinary drugs and vaccines in Mediterranean aquaculture. Options Méditerranéennes: Série A. *Séminaires Méditerranéens*. 86, 41-59. doi:10.13140/2.1.4697.0560.

Scarano, C., Spanu, C., Ziino, G., Pedonese, F., Dalmasso, A., Spanu, V., *et al*. (2014). Antibiotic resistance of *Vibrio* species isolated from *Sparus aurata* reared in Italian mariculture. *New Microbiol.* 37, 329–337.

Siddique, A. B., Moniruzzaman, M., Ali, S., Dewan, M. N., Islam, M. R., Islam, M. S., *et al*. (2021). Characterization of pathogenic *Vibrio parahaemolyticus* isolated from fish aquaculture of the southwest coastal area of Bangladesh. *Front. Microbiol.* 12. doi:10.3389/fmicb.2021.635539.

Snoussi, M., Hajlaoui, H., Noumi, E., Zanetti, S., and Bakhrouf, A. (2008). Phenotypic and genetic diversity of *Vibrio alginolyticus* strains recovered from juveniles and older *Sparus aurata* reared in a Tunisian marine farm. *Ann. Microbiol.* 58, 141–146. doi:10.1007/BF03179458.

Tan, C. W., Rukayadi, Y., Hasan, H., Abdul-Mutalib, N. A., Jambari, N. N., Hara, H., et al. (2021). Isolation and Characterization of Six *Vibrio parahaemolyticus* Lytic Bacteriophages From Seafood Samples. *Front. Microbiol.* 12. doi:10.3389/fmicb.2021.616548.

Urbanczyk, H., Ast, J. C., Higgins, M. J., Carson, J., and Dunlap, P. V. (2007). Reclassification of *Vibrio fischeri*, *Vibrio logei*, *Vibrio salmonicida* and *Vibrio wodanis* as *Aliivibrio fischeri* gen. nov., comb. nov., *Aliivibrio logei* comb. nov., *Aliivibrio salmonicida* comb. nov. and *Aliivibrio wodanis* comb. nov. *Int. J. Syst. Evol. Microbiol.* 57, 2823–2829. doi:10.1099/ijs.0.65081-0

Xu, X., Stern, A. M., Liu, Z., Kan, B., and Zhu, J. (2010). Virulence regulator AphB enhances *toxR* transcription in *Vibrio cholerae*. *BMC Microbiol.* 10, 1–8. doi:10.1186/1471-2180-10-3.

Yuan, Y., Feng, Z., and Wang, J. (2020). *Vibrio vulnificus* Hemolysin: Biological Activity, Regulation of *vvhA* Expression, and Role in Pathogenesis. *Front. Immunol.* 11, 1–8. doi:10.3389/fimmu.2020.599439.

Zhang, X., Lin, H., Wang, X., and Austin, B. (2018). Significance of *Vibrio* species in the marine organic carbon cycle—A review. *Sci. China Earth Sci.* 61, 1357–1368. doi:10.1007/s11430-017-9229-x.

Zorrilla, I., Chabrillón, M., Arijo, S., Díaz-Rosales, P., Martínez-Manzanares, E., Balebona, M. C., et al. (2003). Bacteria recovered from diseased cultured gilthead sea bream (*Sparus aurata* L.) in southwestern Spain. *Aquaculture* 218, 11–20. doi:10.1016/S0044-8486(02)00309-5.
